# Applications of Artificial Intelligence, Deep Learning, and Machine Learning to Support the Analysis of Microscopic Images of Cells and Tissues

**DOI:** 10.3390/jimaging11020059

**Published:** 2025-02-15

**Authors:** Muhammad Ali, Viviana Benfante, Ghazal Basirinia, Pierpaolo Alongi, Alessandro Sperandeo, Alberto Quattrocchi, Antonino Giulio Giannone, Daniela Cabibi, Anthony Yezzi, Domenico Di Raimondo, Antonino Tuttolomondo, Albert Comelli

**Affiliations:** 1 Ri.MED Foundation, Via Bandiera 11, 90133 Palermo, Italy; amuhammad@fondazionerimed.com (M.A.); gbasirinia@fondazionerimed.com (G.B.); 2 Department of Health Promotion, Mother and Child Care, Internal Medicine and Medical Specialties, Molecular and Clinical Medicine, University of Palermo, 90127 Palermo, Italy; domenico.diraimondo@unipa.it (D.D.R.); bruno.tuttolomondo@unipa.it (A.T.); 3 Advanced Diagnostic Imaging—INNOVA Project, Department of Radiological Sciences, A.R.N.A.S. Civico, Di Cristina e Benfratelli Hospitals, P.zza N. Leotta 4, 90127 Palermo, Italy; pierpaolo.alongi@arnascivico.it; 4 Pharmaceutical Factory, La Maddalena S.P.A., Via San Lorenzo Colli, 312/d, 90146 Palermo, Italy; sperandeo@lamaddalenanet.it; 5 Department of Biomedicine, Neuroscience and Advanced Diagnostics (BiND), University of Palermo, 90127 Palermo, Italy; 6 Pathologic Anatomy Unit, Department of Health Promotion, Mother and Child Care, Internal Medicine and Medical Specialties, University of Palermo, 90127 Palermo, Italy; alberto.quattrocchi@unipa.it (A.Q.); giulio.giannone@unipa.it (A.G.G.); cabibidaniela@virgilio.it (D.C.); 7 Department of Electrical and Computer Engineering, Georgia Institute of Technology, Atlanta, GA 30332, USA; anthony.yezzi@ece.gatech.edu

**Keywords:** artificial intelligence, deep learning, machine learning, microscopic images, cell, detection, segmentation, contouring, biomedical fields

## Abstract

Artificial intelligence (AI) transforms image data analysis across many biomedical fields, such as cell biology, radiology, pathology, cancer biology, and immunology, with object detection, image feature extraction, classification, and segmentation applications. Advancements in deep learning (DL) research have been a critical factor in advancing computer techniques for biomedical image analysis and data mining. A significant improvement in the accuracy of cell detection and segmentation algorithms has been achieved as a result of the emergence of open-source software and innovative deep neural network architectures. Automated cell segmentation now enables the extraction of quantifiable cellular and spatial features from microscope images of cells and tissues, providing critical insights into cellular organization in various diseases. This review aims to examine the latest AI and DL techniques for cell analysis and data mining in microscopy images, aid the biologists who have less background knowledge in AI and machine learning (ML), and incorporate the ML models into microscopy focus images.

## 1. Introduction

Cell culture is essential in molecular and cell biology laboratories for studying processes such as the metabolism, physiology, and biochemistry of diseased and wild-type cells [1]. It is widely used to analyze the cytotoxicity of cosmetics, novel chemicals, and drugs on specific cell types [2,3]. In virology, cell culture provides a host environment for viruses to replicate, facilitating the investigation of their development and growth rate [4]. Additionally, it enables the study of specific gene expression by introducing genes into the nucleus of cultured mammalian cells [5,6]. A key advantage of cell culture techniques is the homogeneity and reproducibility of data generated using clonal cell lines, making it a powerful tool for basic research and translational applications [1,7].

Furthermore, cell culture is frequently utilized in the theranostic sector to evaluate radiopharmaceuticals [8], develop novel radiolabeled nanosystems, and discover innovative treatments and diagnostic methods in nuclear medicine [9,10,11]. Analyzing cell characteristics during cultivation is critical and often involves conventional counting plates or semi-automated machines for cell counting, inverted microscopes for observing cell size and morphology, and live cell-tracking techniques for monitoring cellular dynamics over time [12,13,14].

However, cell identification and classification in histopathology and cell culture workflows remain challenging due to their reliance on manual microscopic analysis, which is time-consuming, labor-intensive, and lacks reproducibility [15,16]. The increased application of machine learning (ML) in analyzing microscopic images of cells and tissues is driven by the availability of large volumes of data, cost-effective software and hardware, and the ability to enhance image resolution. These advancements make ML a crucial tool for analyzing the intricate details of microscopic images.

This review aims to assist biologists with little to no background knowledge in ML and artificial intelligence (AI) and incorporate AI and ML models into the microscopic image workflow.

## 2. Material and Methods

We conducted a comprehensive literature search using online databases, including PubMed and Google Scholar. Our objective was to identify studies that applied machine learning techniques to analyze microscopic images of cells and tissues.

The search strategy incorporated the following keywords: (“Artificial intelligence” OR “deep learning” OR “machine learning”) AND (“microscopic images” OR “cells” OR “tissues”).

A time restriction was applied, limiting the search to studies published between 2016 and 2024. Additionally, we manually screened the reference lists of relevant studies to ensure the inclusion of all eligible publications.

### The Inclusion Criteria

Studies were included if they met the following criteria: Utilization of microscopic images of cells and tissues. Segmentation of cellular organelles, including the cytoplasm, nucleus, and cytoskeleton. Classification of live and dead cells, apoptosis, and necrosis. Cell counting and biomarker identification.

Around 20 studies met the inclusion criteria. These studies are summarized in the final section of this review.

## 3. The Basic Concept of Artificial Intelligence Applied in Microscopy Imaging of Cells and Tissues

Machine Learning (ML), a subclass of AI, has emerged as a powerful technique in microscopy imaging [17,18]. It enables biologists to extract meaningful information from cellular and tissue structures with greater speed, accuracy, and reproducibility [17,19,20,21,22,23,24,25,26,27,28,29]. ML utilizes algorithms that learn from the data to identify patterns and features and make predictions without requiring detailed instructions [30,31,32]. ML plays a significant role in tasks such as cell counting, phenotype analysis, and noise correction, including fluorescence microscopy [33,34,35]. Moreover, ML addresses the variability caused by staining techniques like immunohistochemistry (IHC), hematoxylin and eosin (H&E), and fluorescence staining by standardizing image analysis for consistent results. For example, ML algorithms excel at identifying cellular features, delineating irregular boundaries, and classifying cell types in histological samples [36,37,38]. For instance, in histological samples, ML can differentiate cell types, such as stromal, epithelial, or immune cells, by learning features like nuclear morphology and cytoplasmic characteristics.

**Figure 1:**
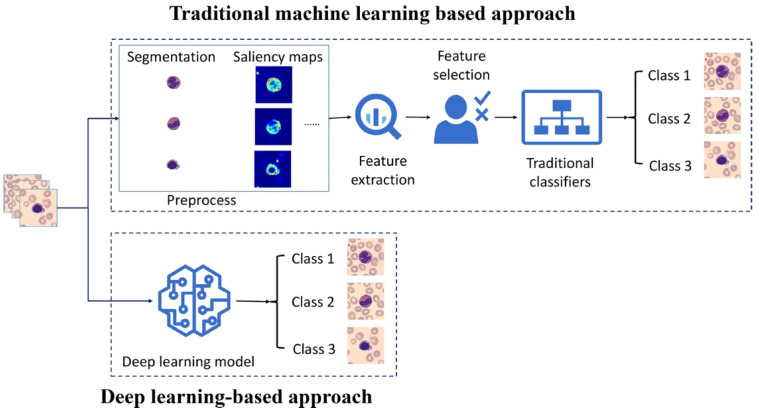
The process of traditional ML and DL approaches. Reprinted with permission from [48].

AI-driven algorithms also facilitate the automated segmentation of cells and tissues, accurate boundary detection, and the classification of different cell types and pathogens, thereby aiding in research and diagnostics (Figure 1) [34,39,40,41,42,43,44].

Moreover, advanced imaging modalities like hyperspectral and multispectral imaging further expand the potential of AI and ML in analyzing microscopic images of cells and tissues [45]. Hyperspectral imaging, which captures information across a wide range of wavelengths, has been used for the optical identification of diabetic retinopathy. It provides detailed spectral and spatial information for an accurate diagnosis [46]. Similarly, multispectral imaging has demonstrated its utility in cancer detection, such as in esophageal cancer, by aiding in spectrum-aided visual enhancements and band selection to improve detection accuracy [47]. These advanced techniques complement traditional microscopy by enabling the analysis of biochemical variations in cells and tissues that might not be visible with conventional methods [45].

The application of AI and ML is particularly significant when studying tissues affected by diseases, such as cancer cells, where cellular heterogeneity is an important factor in understanding disease progression and therapeutic response. By training models, biologists can classify cells or tissues with high accuracy. For example, they can differentiate between healthy and diseased tissue in histopathological samples, identify cellular changes following drug treatments, or uncover a pattern that may not be observed [49,50].

ML can also analyze multichannel images such as fluorescence microscopy to track protein and expression levels [19,51,52,53]. Additionally, ML algorithms can track the movement of individual cells over time in live cell imaging experiments, providing detailed insights into cellular dynamics in response to different conditions or drug treatments [14,54]. In high-content screening, ML is used to analyze large datasets generated by imaging technologies, allowing researchers to discover subtle phenotypic changes in response to genetic or environmental modifications [55,56,57,58]. AI offers numerous advantages in this field, including enhanced accuracy, consistency, time and cost efficiency, and the ability to conduct real-time analysis [59,60,61,62].

ML enables systems to learn and improve their performance from experience without being programmed. It involves the use of algorithms and statistical models to analyze and draw inferences from data patterns [18,63]. ML is categorized into supervised and unsupervised learning. Supervised learning algorithms are trained on labeled data, where the system learns from input–output pairs, making predictions or decisions based on updated data [64]. Common algorithms in this category include linear regression for predicting continuous values, Logistic Regression for binary classification problems, decision trees for decision-making and classification, k-nearest neighbor (KNN) for classification, Discriminant Analysis for classification, and support vector machines (SVMs) for classification tasks [65,66,67]. In contrast, unsupervised learning deals with unlabeled data, where algorithms aim to discover patterns and structures in the data without predefined labels or explicit programming. Techniques in this category include clustering methods such as K-means, Fuzzy C-Means (FCM), and hierarchical clustering, as well as dimensionality reduction techniques like Principal Component Analysis (PCA) [68,69].

Reinforcement learning is a feedback-based learning method in which an agent learns to make decisions by performing actions and receiving rewards or penalties [70]. This method is particularly useful in decision-making tasks like game-playing and robotic control [71]. Advanced ML techniques include DL, which employs neural networks with many layers (deep neural networks) to model complex patterns in data [65]. Convolutional Neural Networks (CNNs) are specialized for processing structured grid data, such as images, and use convolutional layers to learn spatial hierarchies of features from input images [72,73,74]. Recurrent neural network (RNNs), on the other hand, are used for sequential data tasks, like language modeling and time series prediction [75]. Probabilistic Graphical Models (PGMs) provide a robust framework for encoding probability distributions over complex domains, which are helpful for reasoning under uncertainty, including methods such as Bayesian networks and Markov random fields [76]. Active learning, where the ML algorithm selectively queries the user to label data points with ambiguous predictions, reduces the need for large labeled datasets and improves model performance with minimal human intervention [77].

To evaluate the accuracy and reliability of AI models, matrices are used such as the Dice coefficient and Jaccard Index. These metrics are crucial for evaluating the accuracy and reliability of AI models in segmenting and classifying biological structures. The Dice coefficient, a measure of overlap between predicted and ground truth regions, is particularly useful in assessing segmentation tasks where precise boundary delineation is essential. Similarly, the Jaccard Index provides a robust assessment of similarity between two sets. This makes it a valuable metric for determining the extent of agreement between predicted and actual regions. These metrics are not just performance indicators, but also serve as benchmarks for comparing different AI models and methodologies across studies [78].

These metrics are particularly important in biomedical applications because they directly correlate with clinical utility. For instance, high Dice and Jaccard scores in segmenting pathological regions can enhance disease diagnosis, treatment planning, and the monitoring of accuracy. Moreover, they provide quantitative evidence of the model’s capability to replicate expert-level annotations, thereby fostering trust in AI-driven solutions among clinicians and researchers [79].

However, the lack of standardization in metric usage across studies often impedes meaningful comparisons and generalizability. To address this, it is recommended to adopt a unified evaluation framework that includes a core set of metrics—such as the Dice coefficient, Jaccard Index, precision, recall, and F1-score—tailored to specific biomedical imaging tasks. Additionally, providing detailed descriptions of dataset characteristics, preprocessing steps, and evaluation protocols is essential for reproducibility and transparency. Establishing benchmark datasets with standardized annotations can further facilitate cross-study comparisons and drive advancements in the field. By standardizing performance evaluation, the biomedical imaging community can ensure the development of robust, reliable, and clinically applicable AI models [80].

### 3.1. Introduction of Artificial Neural Networks

**Figure 2:**
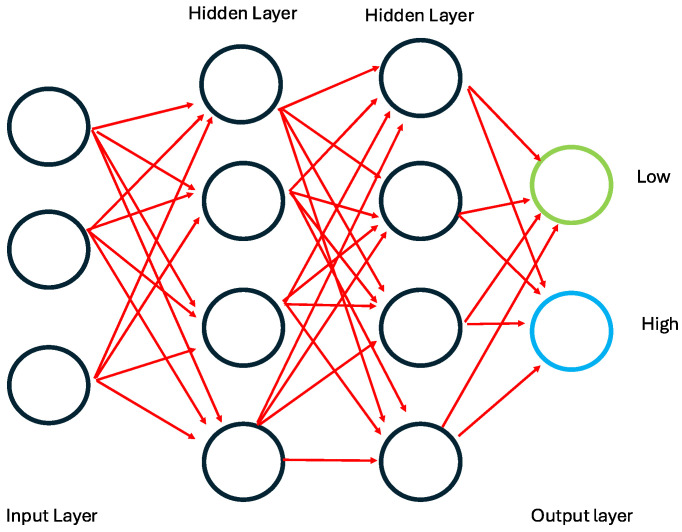
The architecture of deep learning algorithms.

Artificial Neural Networks (ANNs) are computational models inspired by the human brain structure and function [22,81]. They consist of interconnected layers of nodes or neurons, where each connection has an associated weight. The primary components of an ANN include an input layer, one or more hidden layers, and an output layer [82]. Neurons in the input layer receive external data passed through the hidden layers. Each hidden layer applies specific transformations using activation functions to capture complex patterns in the data (Figure 2). The output layer generates the final predictions or classifications [59].

Training ANNs involves several critical hyperparameters, such as batch size, loss function, and optimizer, which influence model performance and convergence [83]. Batch size refers to the number of training samples passed through the network before updating the model’s weights. Smaller batch sizes can introduce more noise into weight updates, helping the model escape local minima and improve generalization, but at the cost of stability. Conversely, larger batch sizes lead to more stable updates, but risk overfitting and increasing computational demand. The choice of batch size, typically ranging from 16 to 256, depends on the dataset, model architecture, and available computational resources [84].

The loss function is a mathematical tool used to quantify the error between the predicted output and the actual target values during training. For example, in autoencoders—a type of ANN used for tasks like dimensionality reduction and denoising—Mean Squared Error (MSE) is commonly employed as the loss function to minimize reconstruction error. Other loss functions, such as Binary Cross-Entropy, are suitable for specific data types, such as binary or sparse datasets [85,86].

An optimizer is an algorithm that adjusts the network’s weights to minimize the loss function by using gradients computed during backpropagation. Common optimizers include Stochastic Gradient Descent (SGD), which updates weights in small steps and can be noisy, and Adam (Adaptive Moment Estimation), which adapts the learning rate for each parameter, often leading to faster convergence, especially in complex models [87].

ANNs are trained using a process called backpropagation, where the network adjusts the weights of connections based on the error of the predictions compared to the actual outcomes. This process continues until the network achieves a desirable accuracy level [88]. For instance, autoencoders—specialized neural networks—consist of an encoder that compresses input data into a latent space and a decoder that reconstructs the input from this compressed representation. Autoencoders minimize reconstruction error using loss functions like MSE, and are widely used in tasks such as data denoising, anomaly detection, and feature extraction [89]. ANNs are versatile and have been applied in various domains, including image and speech recognition, natural language processing, and predictive analytics, showcasing their ability to model non-linear relationships and complex datasets [90,91].

#### 3.1.1. Introduction of Convolutional Neural Networks

**Figure 3:**
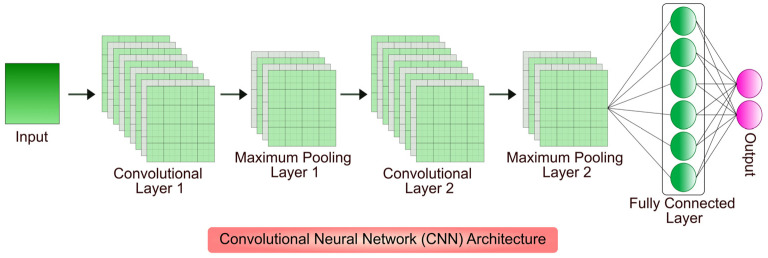
CNN uses multiple convolutional and pooling layers that analyze spatial information. The pooling layer reduces the size of features to enhance computational efficiency, while the convolutional layer applies filters to extract critical features. The fully connected layer then takes the processed data to make the final prediction for the output. Reprinted from [103] under CC BY license.

Convolutional Neural Networks (CNNs) are a specialized type of ANN designed to process structural grid data, e.g., images [92]. CNNs are particularly effective in image recognition and classification tasks because they can automatically and adaptively learn the spatial hierarchies of features [93]. CNN architecture consists of several layers: convolutional layers, pooling layers, and fully connected layers [94]. Convolutional layers apply filters to the input data to create feature maps, capturing local patterns like edges, textures, and shapes [95]. Pooling layers reduce the spatial dimensions of feature maps, decreasing the computational load and controlling overfitting [96]. Common pooling operations include max pooling and average pooling. Finally, the fully connected layers integrate the learned features to make the final classification or prediction (Figure 3) [97]. The convolutional and pooling layers extract high-level features from raw pixel data, enabling CNNs to accurately recognize and classify objects in images [98]. CNNs have revolutionized various fields, including medical image analysis, autonomous driving, and facial recognition, by providing powerful tools for automated image analysis [99,100,101,102].

#### 3.1.2. Introduction of Deep Convolutional Neural Networks

Deep Convolutional Neural Networks (DCNNs) are an advanced form of CNNs that extend their capability by adding multiple convolutional layers, resulting in deep architectures. DCNNs are particularly effective for tasks involving complex data patterns, such as image and video recognition, because they can hierarchically learn and represent data through numerous layers [92,104,105].

**Figure 4:**
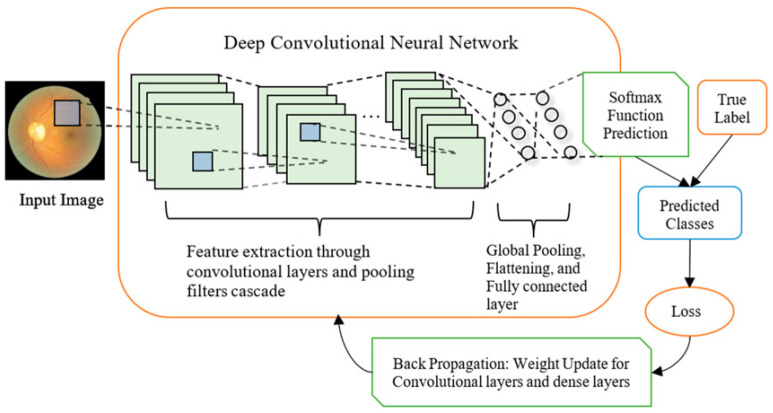
The classification process using deep CNNs (Convolutional Neural Networks) starts with an input image analyzed using convolutional filters. These filters detect patterns across small receptive fields at each image position, producing feature maps. Convolutional or pooling layers process these feature maps until they reach a global average pooling layer, followed by a fully connected layer. The neurons are activated by the ReLU function, and the final layer connects to a Softmax function, which converts the results into probabilities for classifying the input image. During training, the network calculates loss using cross-entropy by comparing predicted classes to actual labels. This loss is backpropagated through the network to update the weights of the filters and fully connected layers, utilizing Stochastic Gradient Descent. Reprinted from [72] under CC BY license.

DCNN architecture typically consists of several types of layers, including convolutional layers, pooling layers, and fully connected layers [106]. Convolutional layers apply filters to the input data to create feature maps, capturing essential features like edges, textures, and shapes. These layers enable the network to detect the spatial hierarchies of features in input images automatically. Pooling layers, such as max pooling or average pooling, reduce the spatial dimensions of the feature maps, decreasing the computational load and helping to control overfitting by making the detection process invariant to minor translations and distortions in the input data (Figure 4) [95].

As the network depth increases, DCNNs can capture more complex and abstract features, making them highly effective at recognizing intricate patterns and objects within images. The fully connected layers at the network’s end integrate the learned features and perform the final classification or regression tasks. The combination of deep layers allows DCNNs to achieve highly accurate tasks such as the classification of images, object detection, and semantic segmentation [107,108,109].

DCNNs have revolutionized many fields, including medical imaging, autonomous driving, facial recognition, and more. Often more accurate than human recognition, they are used for developing systems that can recognize objects in images and videos. Despite their power, DCNNs also have challenges, including the need for large datasets, high computational demands, and difficulties interpreting the learned features. Nonetheless, continuous advancements in network architectures, optimization techniques, and computational power are driving the ongoing evolution and success of DCNNs in various domains [110,111,112,113,114]. A notable example is the use of DCNNs for binary classification tasks in cell biology. For instance, a recent study developed a DCNN to classify human-induced pluripotent stem cell-derived endothelial cells (hiPSC-derived EC) based on photomicrographs. The network incorporated diverse convolutional modules, kernel sizes, pooling layers, and activation functions, achieving high performance with minimal trainable parameters. The model demonstrated high sensitivity, specificity, and precision, highlighting its efficiency in distinguishing cellular features. Additionally, the study introduced an iPS dataset of 16,278 images labeled by expert biologists to facilitate future research. This framework offers an innovative and efficient approach to accelerating and systematizing classification tasks, saving time and effort while enhancing accuracy and consistency [115].

Detecting and segmenting cells in ex vivo tissue makes it feasible to perform high-throughput quantification of various cellular attributes, including cell frequency, morphology, signal intensity, and spatial organization [116,117,118,119,120,121]. Semantic segmentation, or pixel-level classification, is achieved using U-Net or other similarly structured networks in biomedical picture segmentation [122]. U-Net is a deep learning network designed for image processing, particularly useful in scaling down and then up to achieve the exact resolution as the original image with semantic segmentation. Its simple architecture makes it applicable to various medical imaging tasks [123]. Despite being deep, U-Net can be trained quickly and requires low computational resources, evidenced by its training on a GPU RTX 2070 with 8 GB VRAM in approximately one hour. Additionally, U-Net is efficient at prediction, boasting fast-forwarding. However, its main drawback is that it produces binary labels and cannot separate single cells or nuclei by default. In this study, a Keras/Tensorflow U-Net implementation was accessed on 10 January 2022 [124].

Following AlexNet, aDCNN deep learning, which obtained a 15% error rate (nearly 10% better than its competitors) in the ImageNet Large Scale Visual Recognition Challenge, became widely used in imaging sciences [125]. The ramifications of image analysis in medicine were instantly apparent. Deep learning’s use in biological image analysis expanded after several studies investigating DCNNs for use in image analysis were published within a year [126]. Following AlexNet’s success, other DCNN structures have been created for specific medical and biological imaging applications, such as cell segmentation in microscopy and lesion detection in magnetic resonance imaging [34]. DCNNs are not limited to human-defined equations and can identify patterns in unprocessed visual data. Although these patterns might not be understandable to human observers, the network has identified them as the characteristics most helpful in producing reliable and correct conclusions when classifying or segmenting images [127].

The Keypoint Graph Network (KG Network) employs ResNet34-based feature extraction to identify critical points that discretize the input image, processing these key points to extract cell or nuclei bounding boxes, which are then used to derive masks. Unlike U-Net, the KG Network offers object-by-object segmentation, but requires more considerable forwarding time and has a training duration of around four hours on the same GPU. This study utilized a PyTorch implementation (accessed on 10 January 2022 [128].

Mask R-CNN utilizes region-based CNNs to extract cell and nuclei masks. It has a longer forwarding time than the U-Net and KG Networks and demands substantial VRAM for training. It was trained on a cloud node with a GPU NVIDIA K80 with 24 GB VRAM for two hours and thirty minutes. This study used a Tensorflow/Keras implementation of Mask R-CNN (accessed on 10 January 2022), with grayscale images at a resolution of 256 × 256 [129].

#### 3.1.3. Introduction to Generative Adversarial Networks

Generative Adversarial Networks (GANs) are a class of deep learning models that have gained significant attention in recent years for their ability to generate high-quality synthetic data and enhance image analysis tasks [130]. Originally introduced by Goodfellow et al. in 2014 [131], GANs consist of two neural networks—a generator and a discriminator—trained in opposition to each other [132]. The generator learns to create realistic data samples (e.g., images), while the discriminator distinguishes between real and synthetic data, driving the generator to improve its outputs iteratively. This adversarial framework enables GANs to produce highly realistic and complex images, making them a powerful tool for microscopic image analysis [133].

In the analysis of microscopic images of cells and tissues, GANs address several challenges, such as enhancing image quality, mitigating variability in staining techniques, and generating synthetic data to augment training datasets [134,135]. Microscopic imaging often suffers from limitations such as low resolution, noise, or variability in sample preparation (e.g., staining intensity and techniques like immunohistochemistry or hematoxylin and eosin staining) [136,137,138,139,140]. GANs can be leveraged to reconstruct high-resolution images from low-resolution inputs, remove noise, and standardize image quality, improving downstream analyses. For example, super-resolution GANs (SRGANs) have been employed to enhance fluorescence microscopy image resolution, revealing cellular structures with improved clarity [141].

As part of data augmentation, GANs generate realistic synthetic images that mimic the diversity and complexity of biological samples. This is particularly valuable when training machine learning models for segmentation, classification, or feature extraction, where annotated datasets are often limited [130,142,143]. Conditional GANs (cGANs) enable the generation of domain-specific synthetic images by incorporating additional information, such as specific staining methods or cell types, further refining training datasets for targeted tasks [144,145].

Moreover, GANs facilitate cross-modality translation, such as converting fluorescence images into bright-field images or vice versa, reducing the need for multiple imaging modes in experiments. This capability streamlines biomedical research and diagnostics [146,147]. Additionally, GANs have been utilized for domain adaptation, where models trained on synthetic data generated by GANs can be fine-tuned to work effectively on real-world datasets with minimal additional training [148,149].

The integration of GANs into the analysis of microscopic images of cells and tissues has opened new avenues for improving image-based research quality, efficiency, and reliability. By addressing challenges such as data scarcity, noise, and variability, GANs hold promise for advancing cellular biology, histopathology, and drug discovery, among other factors. However, challenges remain in ensuring the generalizability and interpretability of GAN-generated data, which must be addressed for their broader adoption in biomedical research [135,150,151,152,153,154].

#### 3.1.4. Introduction to Vision Transformers

**Figure 5:**
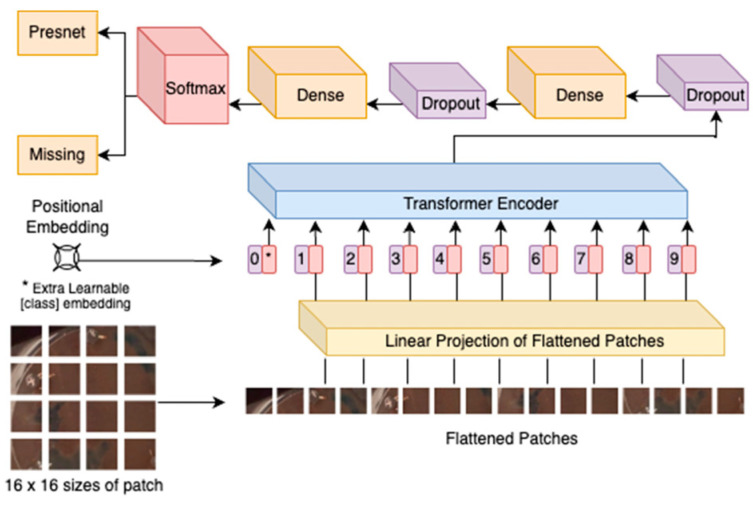
The diagram depicts the Vision Transformer (ViT) architecture for image analysis. The input image is divided into patches (e.g., 16 × 16 pixels), flattened, and linearly projected onto embeddings. Positional and [class] embeddings are added to preserve spatial and global information. These embeddings are processed by a transformer encoder using self-attention to extract features. The output passes through dense layers with dropout and a softmax layer for classification, enabling effective image analysis. Reprinted from [162] under CC BY license.

Image transformers, initially developed for natural language processing (NLP) tasks, have recently demonstrated remarkable performance in computer vision, including the analysis of microscopic images of cells and tissues [155,156,157]. The Vision Transformer (ViT), introduced by Dosovitskiy et al. in 2020, marked a significant shift in image processing by leveraging self-attention mechanisms to model long-range dependencies and complex patterns in image data [158]. Unlike traditional Convolutional Neural Networks (CNNs), which rely on local receptive fields, image transformers operate on image patches, enabling them to capture global contextual information effectively [159,160,161]. The ability to analyze the intricate and diverse features present in microscopic images is particularly suited to their use. ViTs enhance segmentation accuracy in microscopic images of cells and tissues with or without architectures such as U-Net [158]. Figure 5 represents the architecture of ViTs.

Microscopic images of cells and tissues often pose unique challenges, such as high heterogeneity, overlapping structures, and varying scales of biological features [163,164]. Image transformers address these challenges by processing entire image patches simultaneously, allowing the model to analyze spatial relationships and subtle patterns across the entire image [161]. For instance, transformers excel at segmenting cellular components, identifying boundaries between overlapping cells, and detecting subtle morphological changes in tissues. Their ability to generalize across diverse datasets also enhances their robustness in handling variability caused by different imaging modes, staining techniques, or experimental conditions [165].

One of the key advantages of image transformers in microscopy is their scalability to large datasets, which is critical for applications like high-content screening and single-cell analysis [166,167]. By leveraging self-attention mechanisms, transformers can process and analyze high-resolution images without significant loss of detail, making them invaluable for tasks such as cell type classification, subcellular structure identification, and phenotype prediction [168]. Additionally, hybrid models that combine CNNs with transformers have demonstrated superior performance in microscopic image analysis by integrating the strengths of both architectures—local feature extraction from CNNs and global context modeling from transformers [169,170,171,172,173].

Transformers have also shown promise in tasks like image reconstruction and enhancement, which are essential for improving microscopic image quality. For example, denoising transformers remove noise from fluorescence microscopy images. Super-resolution transformers can enhance the resolution of low-quality images, revealing the fine details of cellular structures. Furthermore, transformers have been employed in multimodal analysis, where they integrate information from multiple imaging modalities (e.g., fluorescence and phase-contrast microscopy) to provide a more comprehensive understanding of biological processes [174,175].

Despite their potential, image transformers in microscopic imaging are still an emerging field, with ongoing research addressing challenges such as computational complexity and the need for large, annotated datasets. Nonetheless, their ability to capture intricate spatial relationships and process large-scale data positions them as a transformative tool for advancing cellular and tissue-level research. From diagnostics to drug discovery, image transformers hold the potential to revolutionize how researchers analyze and interpret microscopic images in both academic and clinical settings [161,167,176].

## 4. Experimental Studies Using Artificial Intelligence

In this review, we included studies that demonstrated advancements in AI-based microscopic image analysis, specifically targeting biological models relevant to cell viability, segmentation, classification, and biomarker quantification. Studies were selected based on their use of AI frameworks and application to various cell lines and tissues. Key parameters, such as the aim of the study, the biological model, model framework, statistical evaluation metrics, and dataset availability, were carefully analyzed. A summary table was added (at the end of this section) to provide a clear and comparative overview of the included studies, highlighting trends in AI methodologies, evaluation metrics, and model performance.

**Figure 6:**
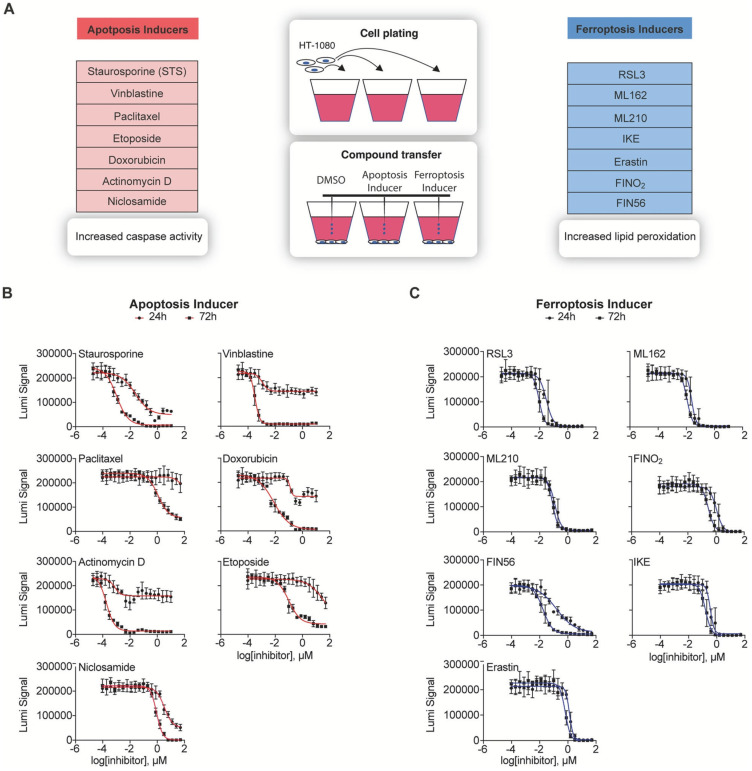
Summary of cell death inducers. (**A**) HT−1080 cells were exposed to apoptosis and ferroptosis inducers, with DMSO as a solvent control. Cells treated with apoptosis triggered apoptosis through caspase activation. In contrast, treatment with ferroptosis inducers led to lipid peroxide accumulation, leading to ferroptosis. (**B**) Viability assay results for HT−1080 cells treated with apoptosis stimulants after 24 h and 72 h incubation. Cellular ATP levels were assessed using luminescence signals. (**C**) Similar results were obtained in B, but for ferroptosis inducers. Reprinted from [177] under CC BY license.

**Figure 7:**
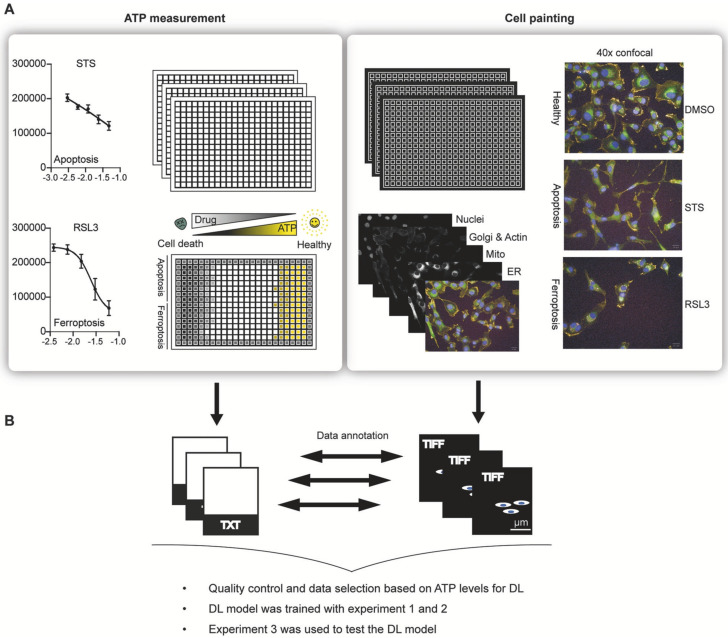
(**A**) HT−1080 cells were exposed to five different concentrations of apoptosis and ferroptosis inducers. Parallel experiments were performed to measure ATP levels (**left**) and conduct cell painting assays (**right**). Staurosporine (STS) and RSL3 were representative examples of apoptosis and ferroptosis inducers, respectively. (**B**) The experimental data is converted into files (TXT) and image files (TIFF). There is a bidirectional exchange of annotated data between these formats. This step is crucial for data annotation, allowing further analysis using AI/ML approaches. Reprinted from [177] under CC BY license.

In one study conducted by Schorpp et al., they developed a DL model known as CellDeathPred to validate and determine the precise classification of different types of cell death, specifically apoptosis and ferroptosis. This model utilized imaging data from the HT-1080 fibrosarcoma cell line, subjected to various inducers and inhibitors (Figure 6). The study focused on the necessity of establishing accurate concentration series for the substances used to generate standardized data, which helps minimize technical variability. Through the cell painting assay, the researchers observed healthy, apoptotic, and ferroptotic cells based on overall cellular content rather than specific markers, enabling clear classification of different cell death modes (Figure 7).

**Figure 8:**
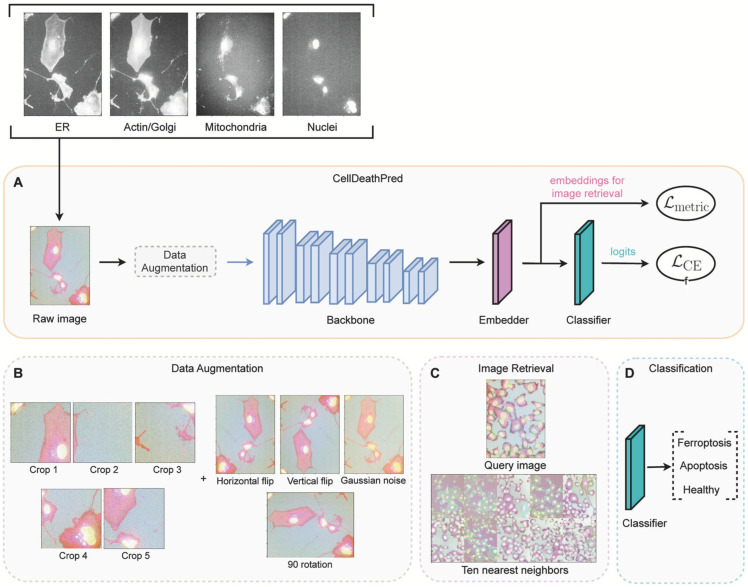
(**A**) The neural network receives four images as input: ER, Actin/Golgi, Mitochondria, and Nuclei. It can predict whether the medication employed in the experiment causes DMSO-induced apoptosis or ferroptosis. The architecture has four stages. A pre-trained network called Efficientnet-b0 is the backbone model. (1) Data augmentation is used to make the model robust during training. (2) A series of fully connected layers called an embedder is applied to create low-dimensional data. (3) A classifier composed of fully connected layers predicts the modality as output. (**B**) Data augmentations applied consisted of 512  ×  512 crops, four corner crops, and one center crop. Amplification was implemented for every crop. (**C**) An illustration of a retrieval image. Ten closest neighbors in the embedding space for a query image. (**D**) The model’s final layer, which has three nodes. Predictions about the classification of the three classes. The orange color represents the deep learning framework, while gray highlights the data augmentation section. Pink indicates image retrieval and embeddings, and blue represents classification outputs. The pinkish cells with a blue-gray background mimic real microscopic images for biological relevance. Reprinted from [178] under CC BY license.

**Figure 9:**
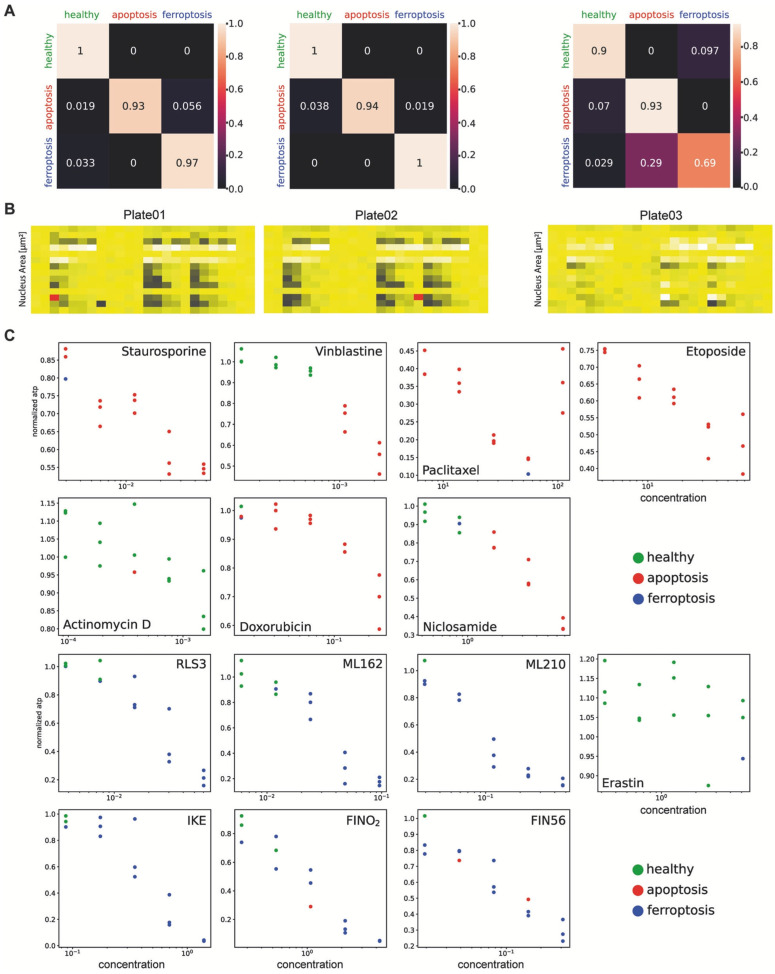
(**A**) Confusion matrix based on the model CellDeathPred for the experiment (non-confocal). The images from the first and second experiments were used to train the model. Plates are arranged from left to right. (**B**) heatmap showing the number of nuclei found in the cell painting experiment photos. Low and high signals indicate the number of selected nuclei. Three plates were used as technical replicates for the experiment. Five different concentrations of each tiny chemical were applied to the cells. (**C**) Prediction of each material based on the ATP level (normalized) at each concentration throughout the plate was conducted using the CellDeathPred model on Plate 01 (non-confocal) for Experiment 3. Three duplicates represent each concentration. Reprinted from [177] under CC BY license.

CellDeathPred integrated contrastive learning with EfficientNet models, leveraging contrastive and cross-entropy losses to achieve high prediction accuracy (Figure 8). The model demonstrated near-perfect classification performance, outperforming traditional machine learning methods like Random Forest, Logistic Regression, and AdaBoost (Figure 9). Future research could explore additional substances and cell death types, such as necroptosis and pyroptosis, to further validate and broaden the model’s applicability [177].

**Figure 10:**
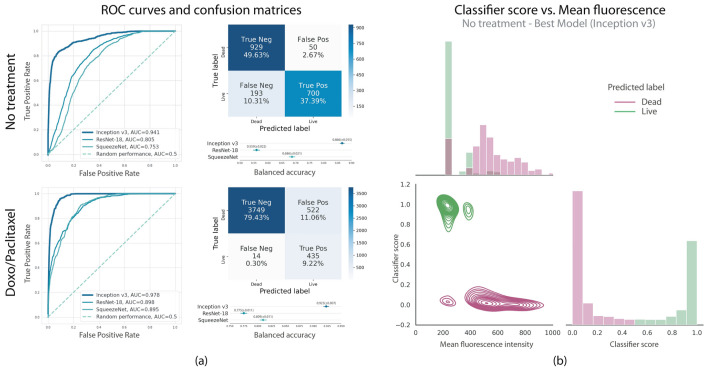
(**a**) ROC curves illustrate the classification performance of each CNN architecture on the testing datasets. The Inception-v3 model demonstrates superior performance compared to ResNET and SqueezeNET. (**b**) Analysis of mean fluorescence versus classifier scores for the top-performing model on the “No treatment” dataset (Inception-v3). Higher mean fluorescence intensities are predominantly associated with lower classification scores, corresponding to dead cells, while lower fluorescence intensities are grouped near higher classification scores, indicating live cells.

In another study, Pattarone et al. conducted a study to determine the live or dead cells of JIMT-1 breast cancer cells using morphological characteristics from bright-field images. The aim was to demonstrate the classification of live and dead cells by utilizing image processing techniques. JIMT-1 cells were treated with chemotherapeutic drugs such as doxorubicin and paclitaxel. In that study, different classifiers were trained through a CNN to perform supervised classification, with labels obtained from fluorescence microscopy images. Figure 10 illustrates the performance of the trained classifiers. All three models demonstrated superior performance compared to random chance on both datasets, successfully extracting relevant image features to classify JIMT-1 cell images as living or dead. Among the models, Inception-v3 achieved the highest performance, with accuracy exceeding 85% across both testing datasets. For the “No treatment” dataset, the balanced accuracy was 0.866 (95% CI: [0.851, 0.881]), with an AUC of 0.941, while for the “Doxo/Paclitaxel” dataset, the balanced accuracy reached 0.923 (95% CI: [0.916, 0.930]), with an AUC of 0.978. Confusion matrices and ROC curves are presented in Figure 10. Additionally, correlations were calculated between the mean values of PI and the classification scores for each image in the testing set to assess the relationship between classification outcomes and fluorescence images. A significant inverse Pearson correlation was observed in both scenarios: “No treatment” (r = −0.705, *p* = 0.024) and “Doxo/Paclitaxel” (r = −0.281, *p* = 0.025). These findings suggest that classification scores are linked to fluorescence levels, highlighting a potential avenue for future research to predict fluorescence images from bright-field images.

**Figure 11:**
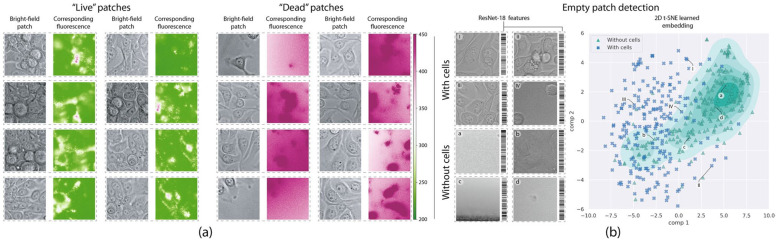
The figure presents a workflow for analyzing cell patches using bright-field and fluorescence microscopy images, focusing on distinguishing between “live”, “dead”, and “empty” patches. In (**a**), representative examples of “live” patches are shown on the left, consisting of bright-field images paired with corresponding fluorescence images highlighted in green, indicating viable cells. Adjacent to these, “dead” patches are displayed with fluorescence images highlighted in magenta, representing non-viable cells. The panel further includes a set of bright-field patches analyzed using ResNet-18 feature extraction, categorized into those containing cells and those without cells. Patches with cells (labeled i–iv) exhibit distinct cellular structures, while patches without cells (labeled a–d) appear largely empty or uniform. (**b**) visualizes the results of an empty patch detection process using a 2D t-SNE learned embedding. Blue stars represent patches containing cells, while green triangles indicate patches without cells. A clear separation is observed, highlighting the model’s capability to distinguish between these two categories. Specific example patches (i–iv, a–d) are mapped onto the embedding space, corresponding to those in (**a**), further validating the effectiveness of the approach in identifying and categorizing microscopy patches based on cell presence.Reprinted from [178] under CC BY 4.0 license.

These results showed the potential of ML and computational image analysis to design new diagnostic tools to reduce costs, save time, and enhance reproducibility in biomedical research (Figure 11). Additionally, the study analyzed how the classifiers grouped bright-field images in the learned high-dimensional embedding and linked these clusters to significant visual characteristics in live–dead cell biology, as observed by trained experts [178].

**Figure 12:**
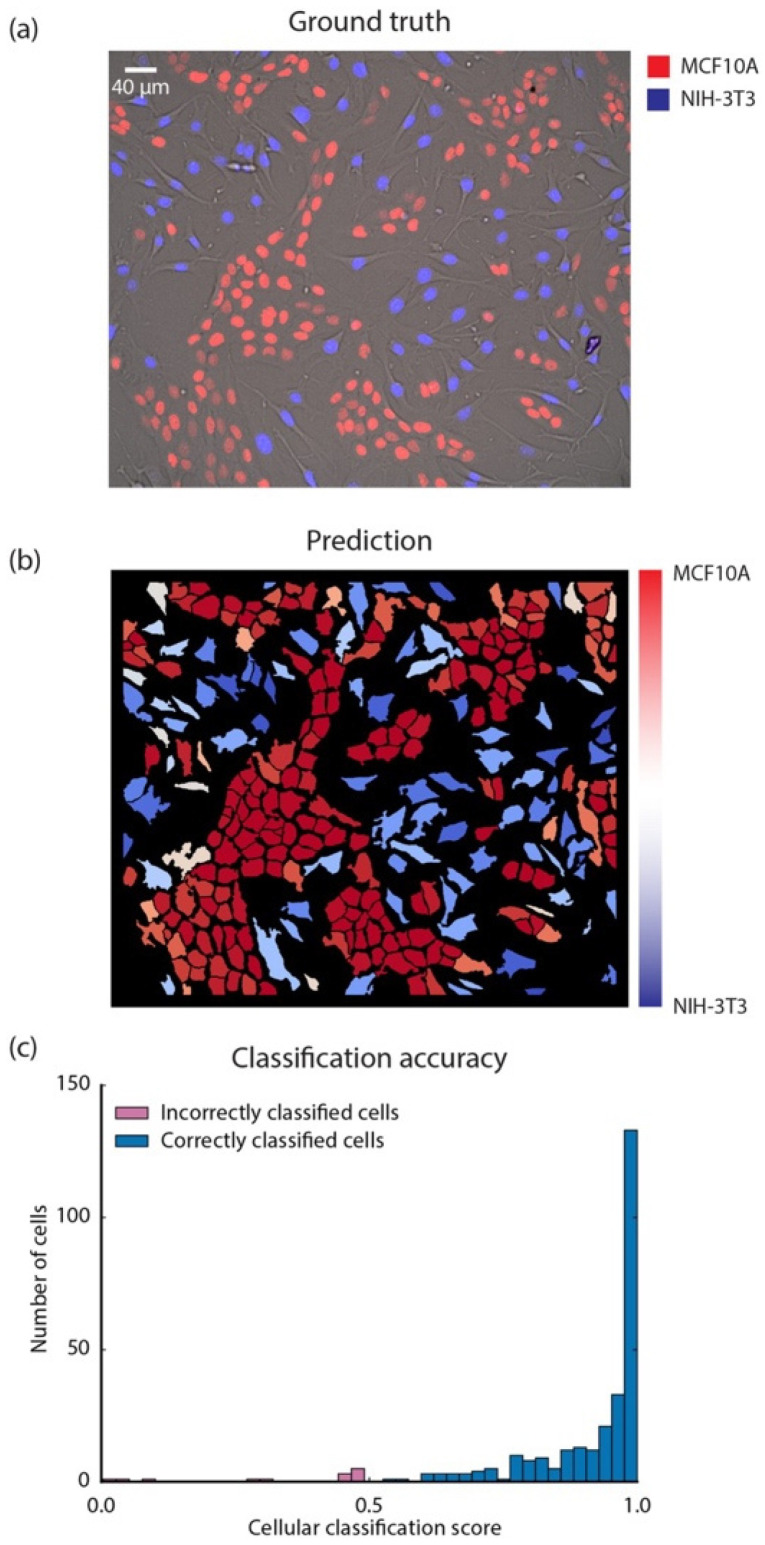
Segmentation and recognition of NIH-3T3 and MCF10A cells using a convolutional neural network (conv-net). The nuclear marker Hoechst 33,342 was implemented as a distinct channel in each image of NIH-3T3 and MCF10A cells to form the training dataset. (**a**) The photograph displays a co-culture of MCF10A and NIH-3T3 cells. MCF10A cells are indicated by an iRFP nuclear marker (red), while NIH-3T3 cells express an mCerulean nuclear marker (blue). An image generated using the Hoechst 33,342 nuclear marker is not included. (**b**) The trained conv-net was utilized to simultaneously segment the image in (**a**) and classify the cell types present. (**c**) Classification accuracy for cell type prediction at the cellular level is illustrated. A histogram depicts the categorization scores for each cell regarding its predicted cell type. The accuracy of predictions is closely linked to the cellular categorization score, with NIH-3T3 cells achieving an accuracy of 86% and MCF10A cells reaching 100% accuracy. Reprinted from [179] under CC BY license.

**Figure 13:**
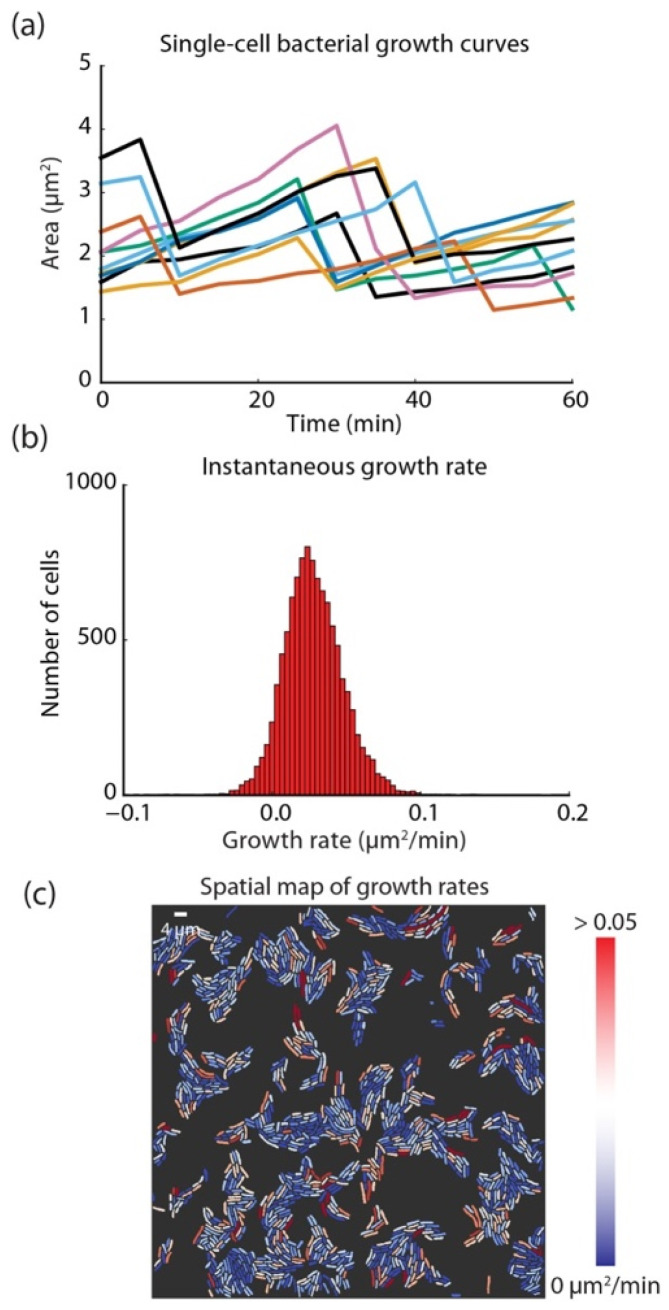
(**a**) *E. coli* single-cell growth curves. The use of conv-nets enables robust segmentation of bacterial cells to create single-cell growth curves from movies of expanding bacterial colonies. For lineage construction, a linear assignment problem-based approach was employed. (**b**) We may create a histogram of the instantaneous growth rate by calculating the area change for each cell from frame to frame. (**c**) Experiments with segmentation masks and instantaneous growth rates can be used to produce a spatial map of growth rates with high accuracy. A map like this makes it possible to identify cells that divide slow or metabolically dormant cells quickly. The different colored lines represent the individual growth curves of single bacterial cells over time. Each line tracks the area (in µm^2^) of a single cell as it changes over a 60-minute period. The variation in color is used to distinguish between different individual cells, emphasizing the heterogeneity in single-cell growth behavior within the bacterial population. These curves highlight that growth is not uniform across all cells; some cells grow steadily, while others show fluctuations or even decreases in size, likely due to measurement noise, division, or other cellular processes Reprinted from [179] under CC BY license.

Another study conducted by Van Valen et al. used different cell types to segment the cytoplasm of both human and bacterial cells using deep CNNs as a supervised ML approach (Figure 12 and Figure 13). They demonstrated that their methodology surpassed existing methods in accuracy, as indicated by the Jaccard Index (JI). The study concluded that deep CNNs are precise and generalizable across various cell types, from bacteria to mammalian cells. Their findings revealed impressive success rates, with a Jaccard Index of 0.95 for bacteria, 0.89 for human nuclei, and 0.77 and 0.84 for various human cytoplasms [179].

In a recent and significant study, immunofluorescence (IF) was effectively conducted on tissue microarrays (TMA) to identify novel biomarkers. The research utilized deep learning AI to automate and enhance the analysis of these biomarkers to predict post-surgical recurrence and metastasis. A total of 648 samples were analyzed, consisting of 424 tumor specimens and 224 normal tissues derived from prostatectomy procedures. The IF staining process incorporated Anti-Ki-67 and ERG antibodies. Analyses were conducted manually and through an AI algorithm that operated independently of clinical data. Manual microscopy assessed the relative mean fluorescence intensity between cancerous and normal tissues. At the same time, the AI algorithm processed digitized images using advanced techniques such as Otsu thresholding, mean shift clustering, and level-set algorithms to delineate cell boundaries accurately. Subsequently, a fully convolutional deep learning model was utilized to refine these identified regions. The results showed that AI analysis of Ki-67 and ERG expression demonstrated only 5% variance compared to manual microscopy. AI achieved 100% accuracy in identifying ERG-positive tumors, even with artifacts. Ultimately, this algorithm exhibited a more efficient, cost-effective, and objective method of analysis, contributing significantly to advancements in the field [180].

In another study, an advanced deep learning-based microscopy method, registration-free GAN microscopy (RFGANM), was used to significantly enhance resolution without requiring additional registration procedures during the training. This method integrates a state-of-the-art GAN (generative adversarial network) to learn how to map low-resolution microscopy images to their high-resolution counterparts. To address challenges associated with complex cellular and tissue patterns, the low-resolution training data are artificially generated and intrinsically registered to the high-resolution training images using a degradation model. This approach simplifies data preprocessing and enhances GAN robustness. Once the model is trained, the AI agent can efficiently reconstruct large field-of-view (FOV), super-resolution images from single low-resolution snapshots captured by standard optical microscopes.

The improved resolution of RFGANM has been validated using imaging resolution targets and PSNR (peak signal-to-noise ratio) analysis, with structural similarity to ground truth samples quantified at over 90%. The method has been demonstrated as a valuable tool for biomedical applications such as cell counting and histopathological diagnoses. It is robust and adaptable to various microscopy methods, including bright-field, epifluorescence, and light-sheet fluorescence images. Notably, it extends the spatial bandwidth product (SBP) of conventional microscopy systems without multiple frames or modifications to the existing hardware.

The RFGANM method combines high resolution and throughput, achieving impressive results such as producing a 0.38-gigapixel digital pathology slide at 1 μm resolution with an acquisition time of 0.01 s and computation time under 1 s. This capability makes it a valuable tool for applications like tissue pathology and neuroanatomy. While minor artifacts in some regions remain and require further refinement through upgrades to the network structure and training algorithms, the method offers excellent performance. Furthermore, although the current demonstration focuses on the 2D imaging of ex vivo samples, the superior spatial–temporal performance suggests its potential for applications in 3D microscopy and highly dynamic biological processes. This innovation bridges the gap between traditional optical microscopy and cutting-edge deep learning techniques, providing a powerful, efficient, and versatile solution for high-resolution imaging in biomedical research [181]

In another study, Multi-StyleGAN, a novel generative adversarial network, simulates time-lapse fluorescence microscopy (TLFM) imagery of living cells as a cost-effective and efficient alternative to traditional TLFM experiments. By leveraging past experimental data, the proposed model synthesizes multi-domain sequences of consecutive timesteps, enabling in silico experimentation for analyzing dynamic cellular processes. The methodology involved training Multi-StyleGAN on a dataset of live yeast cells imaged in micro-structured environments, capturing key biophysical and temporal characteristics such as cell morphology, growth, physical interactions, and fluorescent protein intensity. The results demonstrated that the model effectively generates realistic simulations that encapsulate essential biological properties and temporal dependencies. These simulations have immediate applications, such as generating training and validation datasets for feature extraction algorithms and expediting the development of advanced experimental techniques, including online monitoring and the control of cells [182].

In a recent study, triple-negative breast cancer (TNBC) was identified as the most aggressive form of breast cancer in women, characterized by poor prognosis and limited treatment options. Innovative nano-based carriers are increasingly recognized for their capacity to deliver a range of payloads to cancer cells selectively. Nonetheless, optimizing nanoparticle uptake by tumor cells and ensuring effective drug release are critical considerations in drug development. The study was conducted to evaluate the performance of five CNN models, including two newly developed models and three pre-trained models: VGG16, ResNet50, and Inception-v3. These models were trained using confocal images of TNBC cells treated with nanoparticles containing fluorescent anticancer agents. Comparative and cross-validation analyses were conducted across all models to ensure robustness and reliability. To evaluate the performance of the DL models, their predictions were compared with the conventional method of assessing drug cellular uptake via confocal imaging. A confocal image not previously used for training was selected for this analysis. Using the ImageJ program, manual measurements of average signal intensity across five sample areas yielded a value of 48.5. For reference, the signal intensities from two untargeted nanoparticle confocal images were 33.61 and 17.15, representing significant and low uptake, respectively. Based on these values, the comparison image’s average intensity suggests that the nano-based drug carrier achieved a high cellular uptake. Random patches from the same confocal image were analyzed using the Inception-v3 model, the best-performing DL model in this study. Ten predictions were averaged, resulting in a score of 0.742, with a classification threshold of 0.5 (scores above 0.5 indicating high uptake, and below 0.5 indicating low uptake). These findings corroborate the experiment’s conclusion that the nano-based drug delivery system in the image demonstrates a high cellular uptake of the anticancer drug.

The results showed that these models accurately predicted drug uptake and release in TNBC cells. This suggests their potential utility in early-stage drug development across various research domains, facilitating precise assessments of cellular uptake and enhancing translational outcomes in clinical practice [183].

In one study, Zeng et al. used RIC-Unet (residual-inception-channel attention–Unet) to tailor nuclei segmentation. RIC-Unet integrates residual blocks, multi-scale features, and a channel attention mechanism for more precise segmentation. Comparative evaluations of The Cancer Genomic Atlas (TCGA) dataset against traditional methods (CP and Fiji), original CNN models (CNN2, CNN3), and standard U-net demonstrate superior performance. Evaluation metrics, including the Dice coefficient, F1-score, and aggregated Jaccard Index, show average improvements for RIC-Unet over U-net: 0.8008 vs. 0.7844, 0.8278 vs. 0.8155, and 0.5635 vs. 0.5462, respectively. Additionally, RIC-Unet achieved third place in the MICCAI 2018 computational precision medicine nuclei segmentation challenge [184].

**Figure 14:**
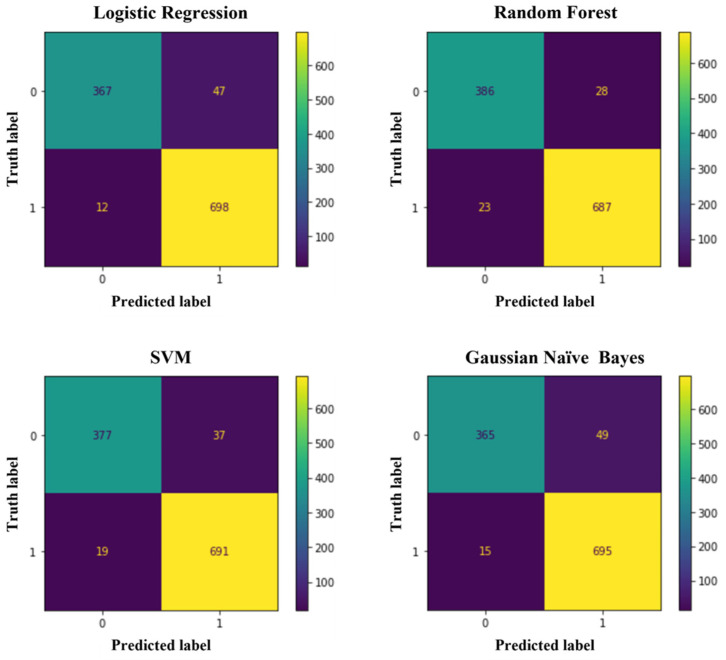
Confusion matrices for the four machine learning models of the cell viability test set. The color bar indicates the number of cells. Reprinted from [185] under CC BY license.

**Figure 15:**
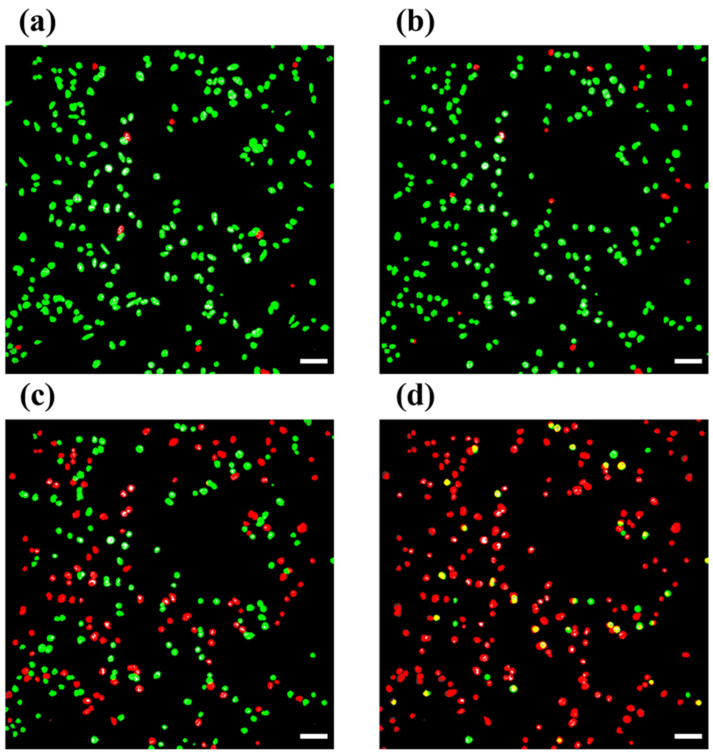
Assessment of cell viability states over time by training a Logistic Regression model after (**a**) 15 min, (**b**) 2 h, (**c**) 4 h, and (**d**) 6 h mark. Green and red labels indicate live and dead cells, respectively. The inset white scale bar represents 100 µm. Reprinted from [185] under CC BY license.

Another study used the ML tool to assess cell viability based on intracellular dynamic activity. The research monitored the fluorescence mean and magnitude of six HeLa cell samples over 24 h using a DFFOCM system. Supervised ML algorithms were trained using these intracellular activity measurements at the 0 h and 24 h marks to distinguish live from dead cells. Despite discrepancies in cell counts due to cell decomposition and detachment, the ML models (Logistic Regression, Random Forest, SVM, and Gaussian Naïve Bayes) achieved a high balanced accuracy of 93.92  ±  0.86% (Figure 14). The study observed a gradual decline in cell viability from 95.59% at 15 min to 16.30% at 6 h, indicating natural cell death in a non-CO2 supplemented environment (Figure 15). ML-based evaluations showed apparent differences from trypan blue staining in assessing cell death, highlighting ML’s advantages in sensitivity and reliability. The study suggests that ML-based analysis of intracellular dynamics could revolutionize clinical trials by predicting cell status and aiding real-time cancer treatment monitoring, particularly for dynamic and malignant cancer cells. The study proposes testing this approach on various cell types beyond HeLa cells, such as breast cancer (MCF-7), lung cancer (A549), and immunotherapy applications (Jurkat cell line), to generalize its applicability. The findings are expected to significantly impact medical research by enhancing cellular analysis capabilities across diverse fields [185].

Another study conducted by Gardner et al. explored the efficacy of CNNs in predicting specific cancer cell lines for label-free identification. The primary objective was to assess whether these CNN models could reliably classify cancer cell lines based on their metastatic potential without chemical labels or the advanced instrumentation typically associated with traditional diagnostic methods. The methodology involved collecting three distinct binary datasets of cancer cell lines, each varying in their metastatic potential. Two CNN architectures, EfficientNetV2 and ResNet-50, were employed for classification, with detailed analysis conducted at each stage of the ML architecture. Training outcomes for each model and dataset were systematically compared, revealing that EfficientNetV2 achieved notable performance, with test set accuracy reaching up to 99%. Moreover, EfficientNetV2 consistently outperformed ResNet-50 across all datasets, showcasing an average accuracy increase of 3.5%. These results demonstrate the potential of DL in enhancing diagnostic precision in cancer research, demonstrating that such models could be effectively retrained and scaled for broader clinical applications in the future [186].

In one study, Lavitt et al. explored two supervised learning strategies for cell counting tasks. The first method involved combining various feature extractors with traditional ML regression models. However, due to the labor-intensive nature and unpredictability of hand-crafted feature extraction, a CNN was employed to treat cell counting as a regression problem, where the image cell count served as a supervised training annotation; deep Residual Network architecture, xResNet, enhanced model performance and leveraged transfer learning from a pre-trained model. The proposed method was evaluated on dense microscope images of two cell lines, human osteosarcoma (U2OS) and human leukemia (HL-60). The results demonstrated superior performance over conventional ML techniques. Moreover, the error margin of our approach (12 ± 15) was comparable to that of a human lab worker. Furthermore, CNN-based regression provided rapid millisecond results, contrasting with the time-consuming nature of human counting processes that span minutes. These outcomes underscore the practical potential of the approach in real-world applications [187].

Another study investigated the structural differences in the actin cytoskeleton between normal and cancer cells, focusing on the human-derived breast epithelial cell line MCF-10A compared to two breast cancer cell lines, MCF-7 and MDA-MB-231. These cancer cell lines differ in their expression of estrogen receptors (ERs), progesterone receptors (PRs), and the human epidermal growth factor receptor 2 (HER2), influencing their invasiveness. The aim was to explore whether these structural differences could serve as diagnostic markers, potentially aiding cancer classification. A CNN-based analysis system was proposed to classify these cells based on their morphological features, achieving superior performance (97.6% accuracy) compared to human experts (78.6%). Transfer learning significantly enhanced CNN performance, with training accuracy reaching 100% on the dataset. However, image enhancement techniques aimed at improving human perception did not improve CNN performance. An analysis of confusion matrices revealed challenges in distinguishing between MCF-10A and MDA-MB-231 cells due to morphological similarities, suggesting that both CNNs and human experts rely on cell morphology for classification. The findings underscore the potential of CNNs in biomedical image analysis, leveraging large datasets to enhance diagnostic capabilities. Future directions could include expanding datasets to include more cell lines and refining segmentation techniques for more precise analysis of intracellular features, aiming to improve classification accuracy and clinical applicability in cancer diagnostics [188].

**Figure 16:**
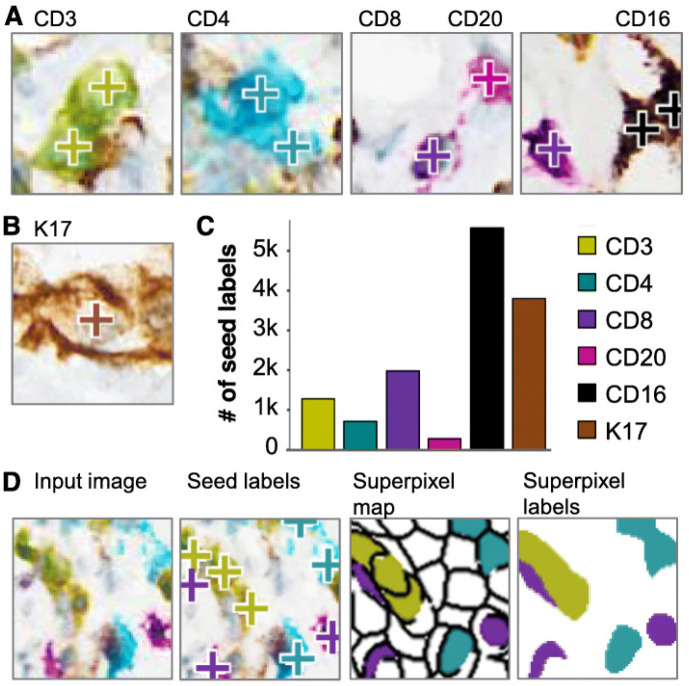
(**A)** Example images of different cell markers identified using multiplex immunohistochemistry (IHC) staining. Each sub-image corresponds to a specific immune marker: Creation of training data for each pixel and seed label annotation of patches. Examples of CD16+ myeloid cells, CD3+, CD4+, CD8+, and CD20+ lymphocytes, and (**B**) K17+ PDAC tumor cells with seed labels superimposed (+). (**C**) The total number of seed labels utilized in all training patches for every cell class. (**D**) The input picture, the input picture overlaid with seed labels, the superpixel map created from the input picture, colored superpixels with varying seed labels, and the superpixel labels used to train the models (based on seed labels and superpixel map). Reprinted from [189] under CC BY 4.0 license.

**Figure 17:**
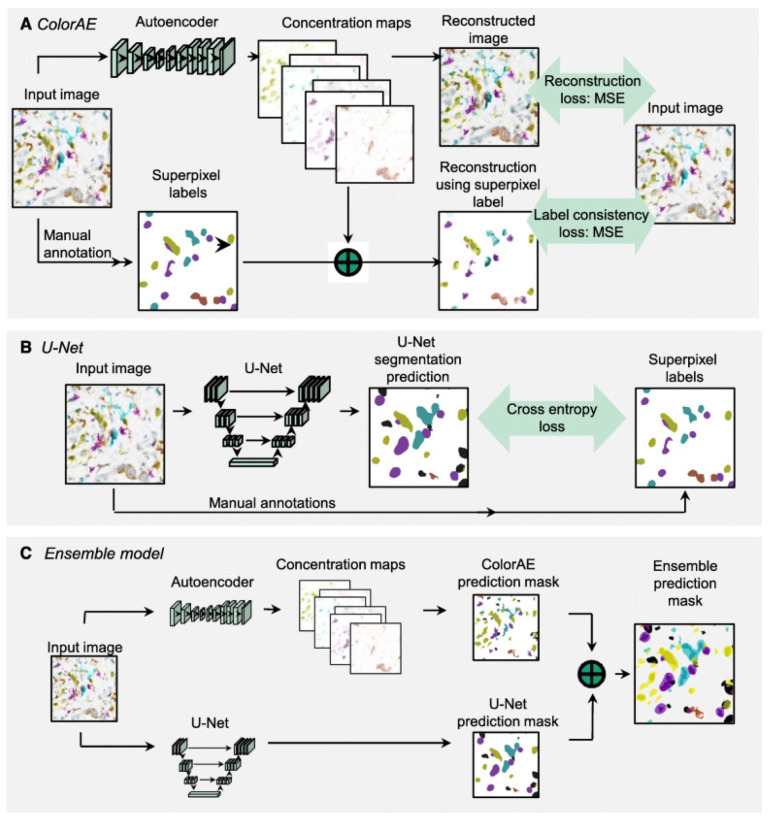
Algorithmic training. (**A**) ColorAE training. Concentration maps of each color (six unique mIHC stain colors: yellow, teal, purple, red, black, and brown; blue hematoxylin nuclear counterstain; and background) are produced by running the input image through an autoencoder. Two loss functions are utilized to guarantee that the rebuilt image includes expert weak annotations and the best integrity to the original image. (**B**) Instruction in U-Net. The input image was processed using U-Net. To optimize the reliability of superpixel labels obtained from hand annotation of the input image, the cross-entropy loss function was utilized—workflow of the ensemble method (**C**). As mentioned, the input image was sent through the autoencoder and U-Net to produce predictions. Reprinted from [189] under CC BY 4.0 license.

**Figure 18:**
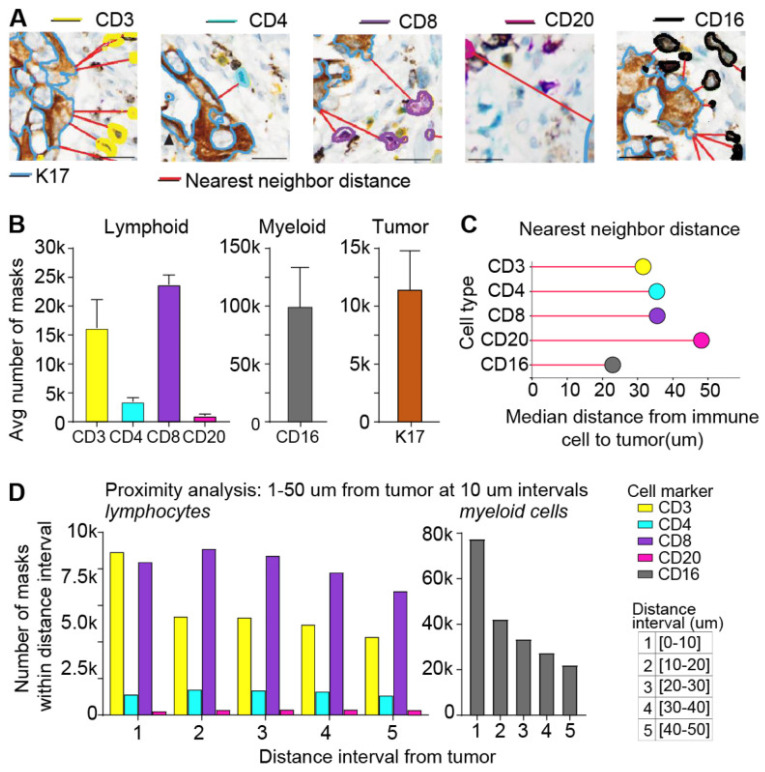
An example of an analysis using mIHC-stained PDAC tissue to show spatial interactions between immunological and cancerous cells. (**A**) Illustrations of the segmentation borders of immune cells and tumor nests have been found, marked with the following IHC biomarkers: CD16, CD3, CD4, CD8, CD20, and K17. (**B**) The mean quantity of masks used in a case across all three WSIs’ cell classes. (**C**) The median distance to nearest neighbors for every kind of immune cell. (**D**) The analysis displays many masks for every cell class spaced 10 µm apart from the tumor’s edge. Reprinted from [189] under CC BY 4.0 license.

In another study, Fassler et al. addressed the challenge of analyzing multiplex immunohistochemistry (mIHC) whole-slide images (WSIs) to quantify the expression of multiple biomarkers across various cell types within pancreatic ductal adenocarcinoma (PDAC) tumor microenvironments (TMEs). Traditional methods for biomarker evaluation in mIHC are often limited and require specialized instrumentation for spectral separation of chromogens. Therefore, this study aimed to develop and validate DL-based approaches to accurately detect and classify six distinct cells labeled with different chromogens in PDAC tissue sections. Methodologically, the study utilized six biomarkers (CD3, CD4, CD8, CD20, CD16, and K17) labeled with differently colored chromogens (Figure 16). It employed an annotation of pathologists to train and validate three deep learning models: ColorAE for color-based object segmentation, U-Net for cell segmentation using color, texture, and shape features, and ensemble methods (Figure 17). Results showed that ColorAE performed comparably to traditional methods for single-stain IHC images. Both ColorAE and U-Net demonstrated robust detection and classification of cell populations, with ensemble methods outperforming individual models (Figure 18). This advancement enabled detailed spatial analysis of immune cell distributions within the PDAC TME, highlighting the potential of deep learning in enhancing the precision and clinical relevance of mIHC analysis in cancer research and potentially clinical applications [189].

In another study, a novel method for nuclei detection was performed by segmenting hematoxylin and eosin (H&E)-stained tissue images, a critical task in clinical and biomedical research. This task is inherently challenging due to significant variations in nuclear staining, size, overlapping boundaries, and the clustering of nuclei. While CNNs have been widely used for such applications, this study explores the potential of transformer-based networks, introducing a Vision Transformer-based architecture called CellViT. The proposed CellViT model is designed for automated cell nuclei segmentation in digitized tissue samples. It was trained and evaluated on the PanNuke dataset, one of the most challenging benchmarks in the field. The dataset comprises nearly 200,000 annotated nuclei across 19 tissue types and 5 clinically important classes, making it a robust and diverse dataset for model evaluation. CellViT leverages large-scale pre-trained Vision Transformers to enhance its performance. The model integrates the recently published Segment Anything Model and a Vision Transformer encoder pre-trained on 104 million histological image patches. This pre-training strategy enables CellViT to effectively capture complex features and patterns in histological images. The model achieved state-of-the-art performance on the PanNuke dataset, demonstrating a mean panoptic quality score of 0.50 and an F1-detection score of 0.83. These results underscore the effectiveness of transformer-based architectures for nuclei detection and segmentation, surpassing conventional CNN-based methods. By addressing the challenges of nuclear variability and clustering, CellViT shows promise as a powerful tool for histopathological analysis, offering significant advancements for clinical applications such as disease diagnosis, prognosis, and research in tissue pathology [190].

**Figure 19:**
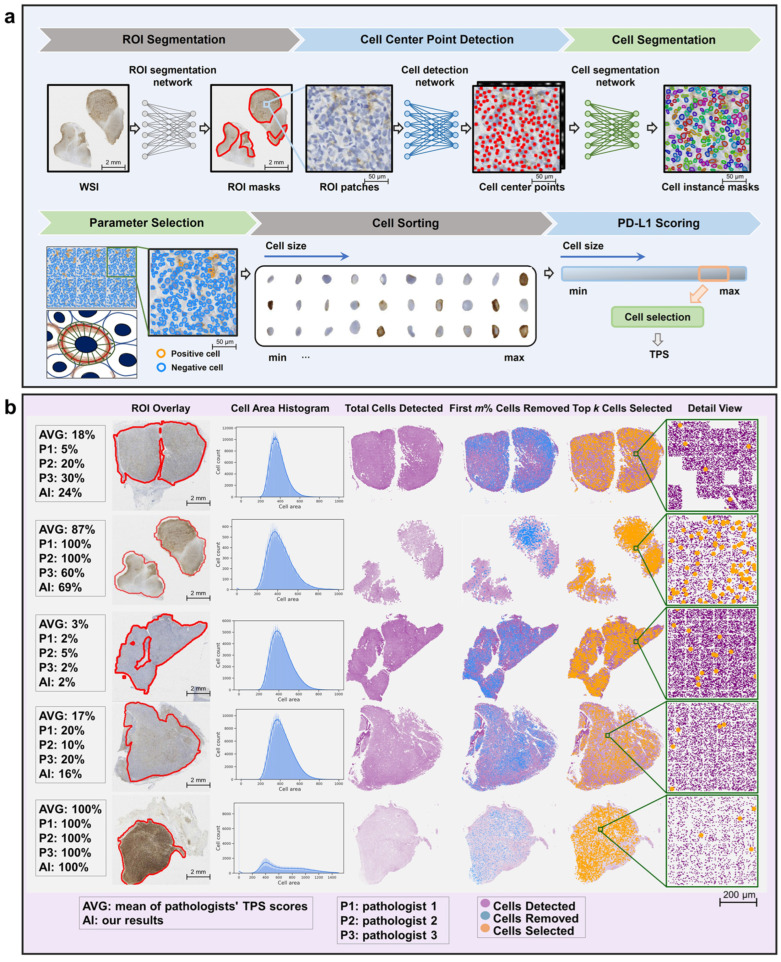
(**a**) The workflow consists of ROI segmentation, cell detection and segmentation, parameter selection, cell sorting, and PD-L1 scoring. (**b**) Quantitative results and a visual representation of the cell distribution, utilizing the suggested immunohistochemistry quantitative rule, are demonstrated. Reprinted from [191] under CC BY 4.0 license.

**Figure 20:**
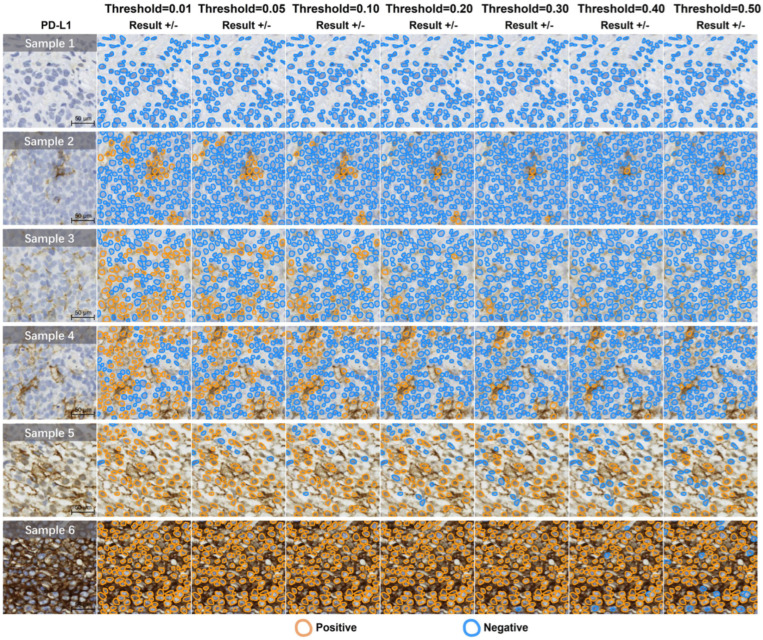
PD−L1 positive expression increases progressively across samples 1 to 6. Reprinted from [191] under CC BY 4.0 license.

In another study, the researchers addressed the critical challenge of accurately quantifying PD-L1 expression in diffuse large B-cell lymphoma (DLBCL) using AI techniques. The aim was to develop an AI-enabled approach that could reliably identify and quantify membrane-positive tumor cells expressing PD-L1, offering an objective tool for pathologists. Methodologically, the study utilized deep learning models for cell detection and segmentation, particularly focusing on whole-slide images (WSIs) from fine needle biopsies and surgical specimens from DLBCL patients (Figure 19). Results demonstrated that the AI models achieved robust PD-L1 quantification performance, providing quantitative results that correlated well with pathologists’ assessments. The study highlighted the efficacy of an integrated pipeline for cell detection and segmentation, emphasizing the ability of AI to streamline and enhance the accuracy of PD-L1 expression analysis (Figure 20). The AI algorithm exhibited higher consistency with pathologists when evaluating samples obtained from fine needle biopsies compared to surgical specimens. This observation is consistent with findings from the primary cohort. The intra-pathologist concordance for fine needle biopsies was reported as 0.97 (95% CI, 0.95–0.98). The intraclass correlation coefficients (ICC) between the algorithm’s outputs and the mean and median scores of pathologists were both 0.96 (95% CI, 0.93–0.98 and 0.93–0.97, respectively), demonstrating substantial agreement. In contrast, the intra-pathologist concordance for surgical specimens was slightly lower at 0.96 (95% CI, 0.92–0.98). The ICC values for surgical specimens, comparing the algorithm’s outputs with the mean and median pathologist scores, were approximately 0.94 (95% CI, 0.87–0.97 and 0.88–0.97, respectively). For fine needle biopsies, the intra-pathologist concordance was notably higher at 0.98 (95% CI, 0.96–0.99). Furthermore, a robust correlation was identified between the AI algorithm and pathologists’ assessments, with ICC values for the mean and median scores reaching 0.98 (95% CI, 0.95–0.99) and 0.97 (95% CI, 0.95–0.99), respectively.

Notably, the research underscored the clinical relevance of AI-driven quantification in aiding immunotherapy decision-making for DLBCL patients, offering a standardized and reproducible approach compared to traditional subjective methods. Despite promising outcomes, the study acknowledged limitations such as the need for further validation on larger, diverse datasets and the ongoing challenges in dataset availability and model robustness. The integration of additional immunohistochemical biomarkers and the refinement of AI models represent promising avenues for future research to advance diagnosis and treatment strategies in lymphoma patients [191].

Another study’s aim was to establish a workflow for detecting ICOS (Inducible T-cell COStimulator) protein expression in colorectal cancer (CRC) using deep learning techniques. The problem addressed the need for the accurate and reliable detection of ICOS-positive cells, which is crucial for understanding immune responses in CRC. Methodologically, the study explored various deep learning architectures, evaluating different pre-trained backbones, batch sizes, loss functions, and training dataset sizes to optimize ICOS cell detection. The results indicated that pixel-level segmentation using the U-Net architecture with the EfficientNetB7 backbone, Adam optimizer, BCE (Binary Cross-Entropy) loss function, and a batch size of 8 yielded the highest performance metrics compared to object-level approaches. Comparison with ground truth annotations provided by pathologists demonstrated close alignment after post-processing, affirming the model’s accuracy in ICOS-positive cell detection. Additionally, the study applied density estimations to validate ICOS expression patterns, confirming robust results with high correlation coefficients. Notably, the developed workflow extended beyond technical validation, showcasing the model’s utility in predicting survival outcomes for stage II/III CRC patients. This application exemplifies how deep learning models can advance beyond segmentation tasks to develop prognostic and predictive tools, leveraging robust clinical data integration for enhanced clinical decision support in oncology [192].

**Figure 21:**
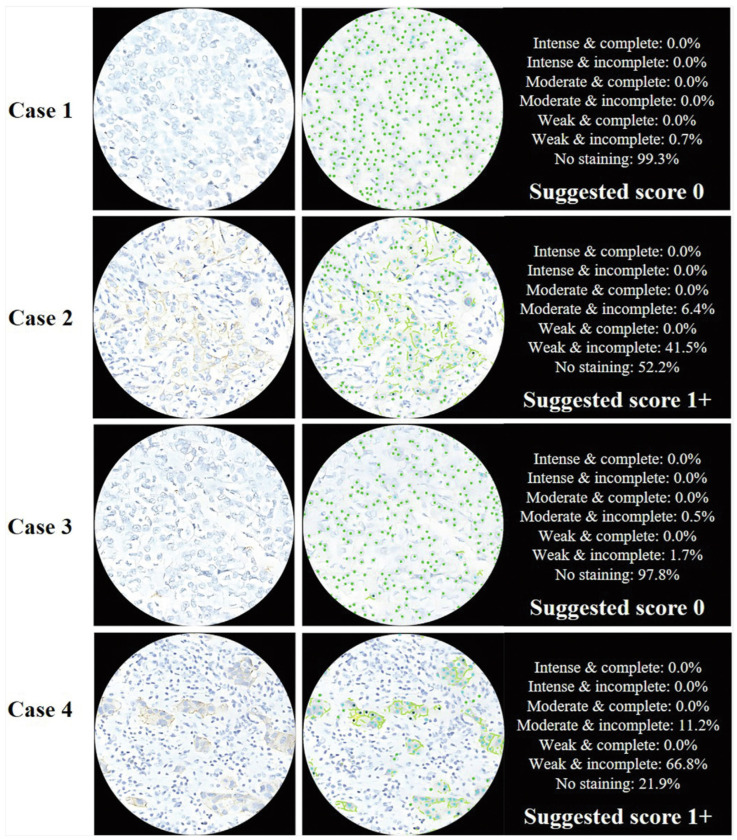
HER2 images were interpreted with AI assistance and under a traditional microscope. Reprinted with permission from ref. [193].

**Figure 22:**
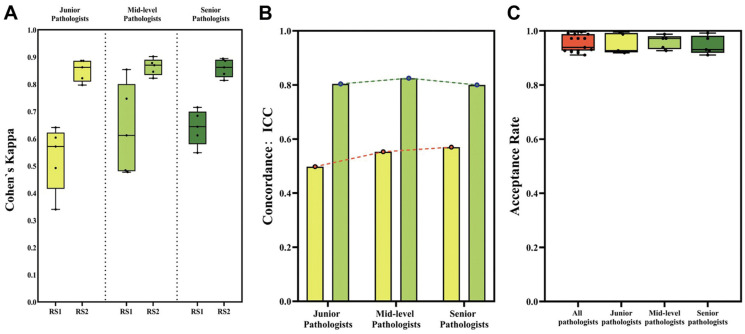
The concordance, acceptance rate, and accuracy of pathologists at various levels. (**A**) A comparison of the accuracy of the two ring studies’ (RSs) junior, mid-level, and senior pathologists. (**B**) Pathologists’ intra-observer concordance varies between RS1 (yellow) and RS2 (green). (**C**) The percentage of pathologists at various levels who accept AI results. ICC is the intraclass correlation coefficient and AI stands for artificial intelligence. Reprinted with permission from ref. [193].

In one study, the researchers explored the role of AI in improving HER2 interpretation accuracy and consistency, especially in cases with heterogeneous expression patterns. The study involved selecting 246 consecutive real cases and conducting a two-round evaluation using an AI-assisted approach. Various aspects of AI models, including pre-trained backbones, batch sizes, loss functions, and dataset sizes, were investigated to optimize the detection and classification of HER2 0 and 1+ tumors (Figure 21). The results demonstrated that AI-assisted interpretation significantly increased the precision and recall for distinguishing HER2 0 from HER2 1+ tumors compared to assessments by pathologists alone (Figure 22). The AI models, particularly effective in identifying faint or barely perceptible HER2 staining, achieved high accuracy even in cases with HER2 heterogeneity, common in HER2 1+ and 2+ tumors. The study also highlighted improvements across all levels of pathologist experience, with junior pathologists benefiting the most from AI assistance initially and mid-level pathologists achieving optimal accuracy after AI integration in the second round. The study outcome supports the implementation of AI-based technologies to supplement the evaluations of pathologists, thereby enhancing the reliability of HER2 IHC scoring and improving patient selection for HER2-targeted therapies. Despite limitations such as the subjective nature of selecting fields of view and the need for a consensus in defining heterogeneous cases, the study underscores the promise of AI in advancing diagnostic precision and therapeutic decision-making in HER2-low breast cancer management [193].

In one study, a ML model was used to accurately classify distinct cell lineages in digital contrast microscopy images and predict optimal cell quantification. Using a CNN, the study achieved remarkable results, with an overall accuracy of 93% and ROC curve results approaching 1.0, indicating robust performance. However, specific cell lineages such as SH-SY5Y (78%), HUH7_mayv (85%), and A549 (88%) exhibited slightly lower accuracy than others. This study demonstrated not only the effectiveness of the CNN-based model, but also its ability to meet microscopic image analysis challenges. In conclusion, this study demonstrates the effectiveness of machine learning in automating and improving the accuracy of cell identification and quantification processes [194].

In one study, the radiation effect on eukaryotic cells was examined using live cell phase-contrast microscopy combined with AI. The researchers used CeCILE (Cell Classification and Identification with Live cell Evaluation), a DL algorithm designed to classify cells into four morphological states: living, dividing, round, and dead. By using a CNN classification and a faster RCNN object detection model, CeCILE provides a robust approach to single-cell analysis in microscopic videos. The study involved the establishment of a labeled dataset containing two widely used cell lines, CHO-K1 and HeLa, to train and validate the algorithm. Despite challenges such as the limited number of detectable cells and reduced performance for less-represented cell states (e.g., dead and dividing cells), CeCILE demonstrated a high classification accuracy, with an F1-score of 0.93. Moreover, object detection performance achieved a mean Average Precision (mAP) of 91% in scenarios with fewer than 100 cells per frame. The study also compared CeCILE’s results with conventional methods, such as colony-forming assays (CFA) and FACS analysis. While CeCILE successfully captured cell proliferation trends and provided valuable insights into radiation-induced stress responses, it was observed that the algorithm currently lacks the capacity to handle high cell densities or track individual cell lineages. Future enhancements, including improved generalization, tracking capabilities, and extended datasets, aim to address these limitations [195].

In one study, Devkota, L conducted a study to investigate the application of radiomic analysis of nanoparticle contrast-enhanced CT images, termed nano-radiomics, as a noninvasive approach to detecting subtle changes in tumor morphology and the tumor microenvironment (TME) associated with responses to myeloid-derived suppressor cell (MDSC)-directed cellular immunotherapy. Specifically, the study aimed to identify quantitative imaging biomarkers that could serve as early indicators of therapeutic efficacy in targeting immunosuppressive components of the TME, such as MDSCs, in solid tumors. This was motivated by the limitations of conventional imaging metrics and the need for more sensitive tools to assess the efficacy of TME-directed therapies during clinical trials. The findings of the study indicate that MDSC-directed cellular immunotherapy using NKG2D ζ-modified NK cells effectively reduces intertumoral MDSC levels, lowers microvessel density (MVD), and modifies the immunosuppressive tumor microenvironment (TME) by decreasing the levels of suppressive cytokines such as IL-6, IL-10, and TGF-β. Despite these significant alterations in the TME, the therapy does not result in noticeable tumor size reductions. Radiomic analysis leveraging AI and ML identified distinct nano-radiomic features that differentiate treated tumors from untreated ones, offering a novel approach to evaluating therapeutic outcomes in TME-directed interventions, independent of traditional size-based metrics. These findings emphasize the complexity of tumor responses and the need for advanced imaging-based metrics to assess therapeutic efficacy comprehensively.

This approach addresses the limitations of traditional imaging metrics, offering a pathway for more accurate, noninvasive therapeutic efficacy assessment. The findings also highlight the potential for clinical translation, as these methods can improve treatment monitoring and decision-making for next-generation immunotherapies. Future research should focus on validating these RFs across diverse tumor models, optimizing imaging protocols for clinical settings, and exploring combinatorial therapies to enhance overall treatment outcomes [196]. Table 1 summarizes all AI-based cell analysis studies considered in the manuscript and where biological models, model frameworks, and performance metrics can be compared.

## 5. Conclusions and Discussion

Microscopic imaging of cells and tissues plays an important role in various scientific and medical fields, providing crucial insights at the cellular and molecular levels. Algorithms such as machine learning and artificial intelligence can analyze microscopic images with high accuracy and precision, often exceeding human capabilities. They can detect details and patterns difficult to detect for human eyes. AI and ML enable image analysis automation. They process large volumes of microscopic images in less time, enabling high-throughput analysis that would be impractical or time-consuming for humans. By automating tasks such as image segmentation, feature extraction, and classification, AI and ML reduce human error in microscopic imaging analysis. This leads to more reliable and reproducible results.

However, AI and ML models heavily depend on the quality and diversity of the data used for training and validation. Microscopic image quality, including brightness, contrast, and noise levels, can significantly affect AI prediction accuracy. Staining techniques, such as hematoxylin and eosin (H&E), immunohistochemistry (IHC), and fluorescence staining, introduce additional variability due to inconsistencies in staining intensity, protocols, and reagents. In the same way, variations in image resolution, magnification, and imaging equipment can limit the robustness of AI models. Addressing these challenges requires careful data preprocessing, augmentation, and standardization to ensure models generalize well across diverse datasets.

A key element of assessing the effectiveness of AI models in the segmentation and analysis of cells is the use of standard evaluation metrics and benchmarks. Metrics such as the Dice coefficient, Intersection over Union (IoU), precision, recall, and F1-score are commonly employed to evaluate model performance in segmentation tasks. These metrics help quantify boundary detection accuracy, overlap predicted and ground truth regions, and overall classification performance. Benchmarks like the Data Science Bowl, Cell Tracking Challenge, and public datasets such as BBBC (Broad Bioimage Benchmark Collection) or Panoptic Segmentation datasets provide standardized platforms for comparing models under consistent conditions. Including these metrics and benchmarks in the evaluation of AI techniques ensures transparency, reproducibility, and a clearer understanding of the strengths and limitations of each approach.

AI and ML algorithms analyze images faster than humans, significantly reducing analysis time. This efficiency translates into cost savings, especially in research and clinical settings where time is valuable. AI and ML can uncover hidden patterns or correlations in microscopic images that may not be immediately apparent to human observers. This capability can lead to discoveries and insights in biology, medicine, and materials science. AI and ML can integrate microscopic imaging data with other types of data (e.g., genomic data and clinical data) to provide a more comprehensive understanding of biological processes or disease mechanisms.

In medical applications, AI and ML can analyze individual patient data from microscopic images to tailor treatments and therapies based on specific characteristics, contributing to personalized medicine advancement. This article reviews the advantages and challenges of AI and ML in microscopic imaging, highlighting recent developments, promising applications, and future directions aimed at overcoming the current limitations and maximizing the potential of AI and ML technologies in scientific and medical research.

Despite these advancements, ML and DL methods have notable limitations. For instance, the high dependency on large, annotated datasets can be a significant bottleneck. Preparing such datasets is labor-intensive and often requires expert-level annotations. Additionally, many AI models are prone to overfitting when trained on small datasets or faced with data lacking variability. Furthermore, the “black-box” nature of many DL models limits their interpretability, making it challenging for researchers to understand why a specific decision was made by the model. The computational requirements of ML and DL algorithms can also be prohibitive, especially for researchers or institutions with limited resources.

Future research directions should focus on developing interpretable AI models, reducing dependency on large datasets by leveraging transfer learning and self-supervised learning approaches, and improving data augmentation techniques to handle variability in imaging conditions. Moreover, efforts to standardize imaging protocols and create publicly available datasets will enhance model robustness and reproducibility. Collaborative initiatives between biologists, data scientists, and engineers will also be essential in translating AI-based methodologies from research to practical applications in diagnostics and therapeutics.

The advancements presented in CellViT and Multi-StyleGAN open promising avenues for future exploration at the intersection of computational biology and histopathology. Nuclei detection and segmentation can be enhanced using transformer-based networks, which can enhance the interpretation of complex tissue structures and improve the accuracy of diagnostics in clinical settings. Meanwhile, the ability of Multi-StyleGAN to generate realistic time-lapse fluorescence microscopy imagery provides a cost-effective alternative to traditional experimentation, enabling the simulation of dynamic cellular processes and facilitating the generation of synthetic datasets for training machine learning models. Future research could focus on integrating these technologies to create comprehensive frameworks that not only automate the analysis of histological images, but also simulate cellular behavior in real time, ultimately fostering a deeper understanding of cell dynamics and improving patient outcomes through more effective disease diagnosis and prognostic tools.

Ethical considerations must also be addressed when implementing AI in sensitive biomedical applications. Issues such as data privacy, informed consent, and the secure handling of patient data are critical, particularly when working with personal or clinical information. The implications of automated analysis in decision-making, especially in medical diagnostics, raises questions about accountability, bias, and fairness. For example, biases in training data could lead to inaccurate predictions, disproportionately affecting certain populations. Additionally, reliance on automated systems without proper oversight may lead to errors with significant consequences in healthcare settings. Including these ethical considerations ensures that AI and ML technologies are deployed responsibly and equitably.

## Figures and Tables

**Table 1:** Summary of AI-based cell analysis studies: biological models, model frameworks, and performance metrics.

Study	Aim of the Study	Biological Model	Model Framework	Statistical Evaluation Metrics	Model and Dataset (Accessed on 20 December 2024)
Schorpp et al. [177]	Differentiate apoptosis and ferroptosis	Ht-1080 cells	VGG-19	Accuracy = 95% on confocal dataset	https://github.com/peng-lab/CellDeathPred/tree/main/Code https://zenodo.org/records/8375591
Pattarone et al. [178]	Determine live or dead cells	JIMT-1 cells	Inception-v3	AUC = 0.941 for classifying cancer cells without treatment#breark#AUC = 0.978 for classifying cancer cell under drug treatment	https://github.com/emmanueliarussi/live-dead-JIMT-1 https://github.com/emmanueliarussi/live-dead-JIMT-1?tab=readme-ov-file#live-dead-jimt-1-image-data
Van Valen et al. [179]	Segmentation of cytoplasm	NIH-3T3 and MCF10A cells	Conv-net	Accuracy = 86% and 100%	https://simtk.org/frs/?group_id=1052 Private
Claire et al. [180]	Automate and speed up the analysis of numerous biomarkers	Prostate cancer cells	FCN	Accuracy = 100%	Private Private
Zhang et al. [181]	Enhance resolution without requiring additional registration procedures during training	BPAECs	GAN	PSNR = 27.79, SSIM = 0.93#breark#Pathological Section: PSNR = 25.60, SSIM = 0.92	Private Private
Rafia et al. [183]	Selection of effective drug candidate	Triple negative breast cancer cells	VGG16, ResNet50, and Inception-v3	Accuracy for VGG16 = 97.282 #breark#ResNet50 = 97.336 #breark#Inception-v3 = 99.348	Private Private
Zeng et al. [184]	Segmentation of Nucleus	WSIs	RIC-Unet is compared with two traditional segmentation methods: CP and Fiji, two original CNN methods: CNN2 and CNN3, and original U-net	Dice coefficient, F1-score, and aggregated Jaccard Index show average improvements for RIC-Unet over U-net: 0.8008 vs. 0.7844, 0.8278 vs. 0.8155, and 0.5635 vs. 0.5462	Private Private
Park et al. [185]	Assess cell viability based on intracellular dynamic activity	HeLa cells, MCF-7, and A549 cells	Mask R-CNN	Accuracy = 93.92 ± 0.86%	Private Private
Gardner et al. [186]	Expression level of EpCAM	PC-3 cells and DU 145 cells	EfficientNetV2 and ResNet-50	Accuracy = 99% #breark#EfficientNetV2 consistently outperformed ResNet-50 across all datasets, showcasing an average Accuracy increase of 3.5%	Private Private
Lavitt et al. [187]	Cell counting	U2OS and HL-60 cells	Deep Residual Network architecture, xResNet	Error margin = 12 ± 15 comparable to that of a human lab worker	https://github.com/falkolav/cell-counter?tab=readme-ov-file https://zenodo.org/records/4428844
Oei et al. [188]	Cell classification based on actin cytoskelton	MCF-10A, MCF-7, and MDA-MB-231 cells	VGG-16	Accuracy = (97.6% vs. 78.6%) (the superiority of CNNs over humans in performing the task)	Private https://figshare.com/s/6968a38297926c0078c3
Fassler et al. [189]	Quantify expression of multiple biomarkers	PDAC cells	U-Net	Dice coefficient = 0.457 to 0.769	Private Private
Horst et al. [190]	Segmentation of Nucleus	WSIs	U-Net-shaped encoder–decoder network	PQ = 0.50#breark#F1 = 0.83	https://github.com/TIO-IKIM/CellViT?tab=readme-ov-file https://warwick.ac.uk/fac/cross_fac/tia/data/pannuke
Yan et al. [191]	Expression of PDL-1	B-cell Lymphoma	Densenet121, Resnet18, and Vision Transformer (ViT)	ICC = 0.98 (95% CI, 0.95 and 0.99) and 0.97 (95% CI, 0.95 and 0.99)	Private Private
Sarker et al. [192]	Cell segmentation/detection in IHC slides quantifying nuclear staining biomarkers	T-cells	U-Net model, Detectron2 using the backbone of ResNet101 with Adam optimizer, loss function of BCE	The U-Net model achieves the best performance #breark#Accuracy = 98.93% Dice coefficient = 68.84%#breark#AJI = 53.92% #breark#Detectron2 yields the best performance of SPE = 99.63% #breark#ResNet50 with SGD optimizer, BCE+L1, and the batch size of 4	Private Private
Wu et al. [193]	Increase the interpretation accuracy and consistency of HER2 IHC 0 and 1+ evaluation	HER2 IHC cells	U-Net	Localization performance F1 = 0.927 and segmentation performance Dice coeffieneinet = 0.914	Private Private
Ferreira et al. [194]	Cell counting	SH-SY5Y, Huh7, and A549 cells	CNN	Accuracy = 93%, with ROC curve results nearing 1.0	https://zenodo.org/badge/latestdoi/701446984
Rudigkeit, S et al. [195]	Cell Classification: living, dividing, round, and dead	CHO-K1 and HeLa cells	ResNet101	Accuracy with an F1-score of 0.93 #breark#Object detection performance mean mAP = 91% in scenarios with fewer than 100 cells per frame	Private Private
Reich et al. [182]	Synthesize sequences of multi-domain TLFM imagery of multiple yeast cells in micro-structured environments	Yeast cells	Multi-StyleGAN	IS = 1.864 and 2.437 for the BF and GFP channel, respectively	Private Private

**AUC** = accuracy under curve, **PSNR** = peak signal-to-noise ratio, **SSIM** = structural similarity, **PQ** = panoptic quality, **ICC** = intraclass correlation coefficient, **AJI** = aggregated Jaccard Index, **FCN** = fully convolutional network, **SPE** = specificity, **SGD** = Stochastic Gradient Descent, **BCE** = Binary Cross-Entropy, **mAP** = mean Average Precision, **SH-SY5Y** = Neuroblastoma, **HUH7** = liver cell, **A549** = lung epithelial cell, **PDAC** = pancreatic ductal adenocarcinoma, **JIMT-1cell** = breast cancer cell, **BPAEC** = bovine pulmonary artery endothelial cells, **HeLa cell** = derived from cervical cancer cell, **DU 145** = prostate cancer cell line, **PC-3 cells** = prostate cancer cell line, and **HER2** = human epidermal growth factor receptor 2.
